# The genomic road to invasion—examining the similarities and differences in the genomes of associated oral pre-cancer and cancer samples

**DOI:** 10.1186/s13073-017-0442-0

**Published:** 2017-06-07

**Authors:** Henry M. Wood, Catherine Daly, Rebecca Chalkley, Burcu Senguven, Lisa Ross, Philip Egan, Preetha Chengot, Jennifer Graham, Neeraj Sethi, Thian K. Ong, Kenneth MacLennan, Pamela Rabbitts, Caroline Conway

**Affiliations:** 1 Leeds Institute of Cancer and Pathology, University of Leeds, Leeds, LS9 7TF UK; 2 St James’s Institute of Oncology, St James’s University Hospital, Leeds, LS9 7TF UK; 3 Leeds Dental Institute, Leeds General Infirmary, Leeds, LS2 9LU UK; 4 Department of Oral Pathology, Faculty of Dentistry, Gazi University, Ankara, Turkey; 5 Northern Ireland Centre for Stratified Medicine, University of Ulster, Altnagelvin Hospital, Londonderry, Northern Ireland BT47 6SB UK

**Keywords:** Pre-cancer, Oral cancer, Tumour progression

## Abstract

**Background:**

It is frequently assumed that pre-invasive lesions are simpler precursors of cancer and will contain a limited subset of the genomic changes seen in their associated invasive disease. Driver mutations are thought to occur early, but it is not known how many of these are present in pre-invasive lesions. These assumptions need to be tested with the increasing focus on both personalised cancer treatments and early detection methodologies.

**Methods:**

We examined genomic copy number changes in 256 pre-invasive and invasive samples from 69 oral cancer patients. Forty-eight samples from 16 patients were further examined using exome sequencing.

**Results:**

Evidence of a shared ancestor of both dysplasia and carcinoma was seen in all but one patient. One-third of dysplasias showed independent copy number events. The remainder had a copy number pattern that was similar to or simpler than that of the carcinoma. All dysplasias examined contained somatic mutations absent in the related carcinoma.

Previously observed copy number changes and *TP53* mutations were very frequently observed, and almost always shared between dysplasia and carcinoma. Other gene changes were more sporadic. Pathway analysis confirmed that each patient’s disease developed in a different way.

Examining the numbers of shared mutations and the rate of accumulation of mutations showed evidence that all samples contain a population of sub-clones, with little evidence of selective advantage of a subset of these.

**Conclusions:**

These findings suggest that most of the genomic changes driving oral cancer occur in the pre-cancerous state by way of gradual random accumulation rather than a dramatic single event.

**Electronic supplementary material:**

The online version of this article (doi:10.1186/s13073-017-0442-0) contains supplementary material, which is available to authorized users.

## Background

Malignant tumours have been observed to develop after passing through various pre-cancerous stages. Seminal work has suggested that pre-cancerous cells may need multiple mutations to become invasive, and that these may occur in a specific pattern, or one of a small number of patterns, depending on the cancer type [1, 2]. Recent advances in genomic technology have allowed more detailed analysis of the timing of these events in different cancer types [3, 4]. Sophisticated mathematical models have been developed to infer the evolutionary dynamics of early and developing disease from fully invasive specimens [5, 6].

Pre-cancerous lesions are often difficult to study due to their small size and the feasibility of obtaining tissue. Head and neck squamous cell carcinoma (HNSCC), however, is an excellent disease in which to study pre-cancer, in that invasive carcinoma frequently presents alongside pre-cancerous dysplasia, which is macroscopically visible and easily accessible due to its location. This has enabled several studies to examine groups of dysplasias and carcinomas [7–11]. Fortuitously, HNSCC may be one of the cancer subtypes that would benefit most from study of pre-cancer. The recent head and neck study of The Cancer Genome Atlas (TCGA) [12] showed that HNSCC has a particularly mixed set of genomic abnormalities, with few common driver genes. This suggests that the study of early disease instead may be a useful alternative approach.

One of the main subsets of HNSCC is oral cancer. Oral dysplasia is characterised by the presence of architectural and cytological atypia within the surface epithelium. Several classification and grading schemes are based on the World Health Organization (WHO) 2005 guidelines [13], each with varying predictive ability.

A novel binary system [14] for grading of oral dysplasia has the advantage of helping clinicians make treatment decisions for dysplastic lesions, especially those which would have been categorised as moderate dysplasia in the WHO 2005 classification.

Even though carcinoma is frequently described as arising from a bed of dysplasia, the relationship between dysplasias of various grades to each other and to their associated squamous cell carcinoma (SCC) is poorly understood. The genomic pathways from normal tissue through pre-cancerous stages to fully invasive disease has not been characterised in HNSCC. Work on small numbers of patients shows considerable variety in both the genomic evolutionary relationships and the differentially expressed pathways between HNSCC and their related dysplasias [15, 16].

In this study we attempted to better understand what genomic changes occur along the pathway to invasion, whether common patterns could be observed between patients and whether the view of gradually increasing grade leading to invasive disease would be reflected by genomic changes. We looked at gross genomic copy number changes in dysplasia and SCC samples from over 50 patients, and exome sequencing of a subset of those patients.

## Methods

### Samples

Patients with potential or proven cancerous oral lesions were recruited from the Oral Surgery outpatients’ clinic at Leeds General Infirmary and were anonymised after providing informed consent. Blood samples were obtained at the time of diagnostic biopsy, and formalin-fixed paraffin-embedded (FFPE) blocks were subsequently obtained from the hospital archives.

Following sectioning, marking of areas of dysplasia and SCC (Additional file 1: Figure S1), and dissection, DNA was extracted using the Qiagen QIAamp DNA Mini Kit or QIAamp DNA micro kit. It was noted whether dysplasia samples were high (HGD) or low (LGD) grade, according to the binary grading system [14], and whether they were present in the same FFPE block as the SCC, or a different block, and therefore slightly further away, physically, from the SCC.

### Low coverage copy number sequencing

Low coverage genomic sequence data were obtained for each patient using an updated version of previously published protocols [17]. DNA was prepared for Illumina sequencing using NEBNext multiplex library preparation kits incorporating custom designed tags. Forty libraries were pooled and sequenced together in one 2 × 100 bp Illumina HiSeq2500 lane, giving around 0.15× coverage per sample.

Sequencing reads were trimmed using cutadapt [18]. Genomic copy number data were produced by CNAnorm [19] after first aligning to the human reference genome (hg19) using BWA [20]. Breakpoints were called using DNAcopy [21]. Human papilloma virus (HPV) infection was tested by re-aligning the data to known viral genomes [22].

Tumour cell content was estimated using CNAnorm, in conjunction with histological examination. Adjusted copy number profiles were then compared as groups of similar samples (LGD, HGD, SCC) and between different samples from the same patient. The differences and/or similarities between dysplasias and associated SCCs were noted. The fraction of genome altered was calculated by summing the number of bases of autosomal genomic gain or loss and dividing by the size of the autosomal genome.

### Exome sequencing

A subset of patients was selected for further analysis if their DNA from dysplasia and SCC was of sufficient quality for adequate copy number libraries to be produced, if the copy number data showed evidence of a disrupted genome, and if the samples proved to be HPV negative. All SCC samples had a tumour cell content of over 70%. All dysplasia samples contained 100% dysplastic tissue.

For each patient, the exomes of matching blood, dysplasia and SCC were sequenced using the Sureselect Human all Exome Kit (Agilent) to an average of 125× coverage. Reads were trimmed using cutadapt, aligned to the human genome using BWA and processed using the GATK pipeline [23]. Mutations were called using Varscan2 [24] in somatic mode. Putative variants were initially filtered to remove any with a Varscan somatic score of 10 or below (*p* > 0.1) and any supported by fewer than three reads.

Following mutation calling, the variant allele frequency (VAF) of each mutation was calculated. The VAFs of mutations which were either unique to each sample or shared between dysplasia and SCC were compared both as histograms and dot plots. As mutation calls with a VAF <0.12 were more likely to be caused by sequencing errors or FFPE artefacts [6], these analyses were performed both with and without those calls with a VAF <0.12. The validity of this cutoff was confirmed by analysis of PCR validation of exome data from a similar cohort of previously published HNSCC FFPE samples [15] (Additional file 1: Figure S2).

### Examining neutral tumour evolution and mutation rate

Following the recent work of Williams et al. [6], the VAFs of each sample were used to test for the presence or absence of neutral evolution. High VAF mutations (>0.24) are more likely to be clonal so were removed, as were low values (<0.12), which were more likely to be errors or artefacts. The remaining mutations represent the expanding and diversifying lesion and capture the sub-clonal architecture. The cumulative number of mutations, M(f), was plotted against the inverse of the VAF, 1/f. The R^2^ goodness of fit for each plot was calculated, with values over 0.98 indicating that the sample was following the neutral evolution model.

For those samples following the neutral model, mutation rates were calculated as the gradient of the M(f) against 1/f plots divided by the 30-Mb exome size, and expressed as mutations per base per effective cell division. This was calculated for all mutations and for each mutation type separately.

### Analysing functional variants

To focus on those mutations which were likely to be driving the disease in each patient, mutations were filtered based on cellular frequency and possible effect on protein function The minimum possible cellular frequency of each mutation was calculated using the VAF, adjusted for normal cell contamination and copy number. For instance, in a triploid region of the genome in a sample with 70% tumour cell content, each tumour cell chromosomal copy will contribute 23.3% of the reads, so a mutation in over 50% of cells will have a minimum VAF of 11.7%. The putative effects of each mutation were predicted using the Variant Effect Predictor [25]. Mutations were kept if they were present in over 50% of cells and had a somatic score of over 15 (*p* < 0.05), in either dysplasia or SCC, and if they had a possible effect on protein function, namely: anything affecting a splice site, frameshifts, stop gained or lost, coding deletions or insertions, initiator codon variants, incomplete terminal codon variants, exon elongations, truncation mutations, or missense mutations that were labelled as either possibly or probably damaging or deleterious. If a mutation passed filters in one sample but was present in lower cellular frequency in the paired sample, it was counted as present in both samples.

### Pathway analysis

For each patient, lists of genes passing filters were split into three lists according to whether they were found in dysplasia only, SCC only or shared. These lists were compared to lists of genes from the KEGG [26, 27] database in pathways previously associated with HNSCC: calcium signalling, cell cycle, DNA replication, ERBB signalling, JAK-STAT signalling, MAPK, NOTCH signalling, PI3K, P53 signalling, phosphatidylinositol signalling, VEGF signalling, WNT signalling and hedgehog. Lists of genes from HNSCC exome papers [28], the cancer gene census [29] and TCGA head and neck study [12] were also used.

A more agnostic approach was taken by importing the gene lists into the DAVID functional annotation tool [30, 31], and keeping those gene ontology (GO) terms that were enriched with a *p* value of 0.05 or better.

## Results

### Samples

Data were collected from 69 patients. Fifty-nine patients provided enough DNA from both dysplasia and SCC samples for comparative analysis, totalling 256 samples (Additional file 1: Table S1).

### Copy number patterns

**Fig. 1:**
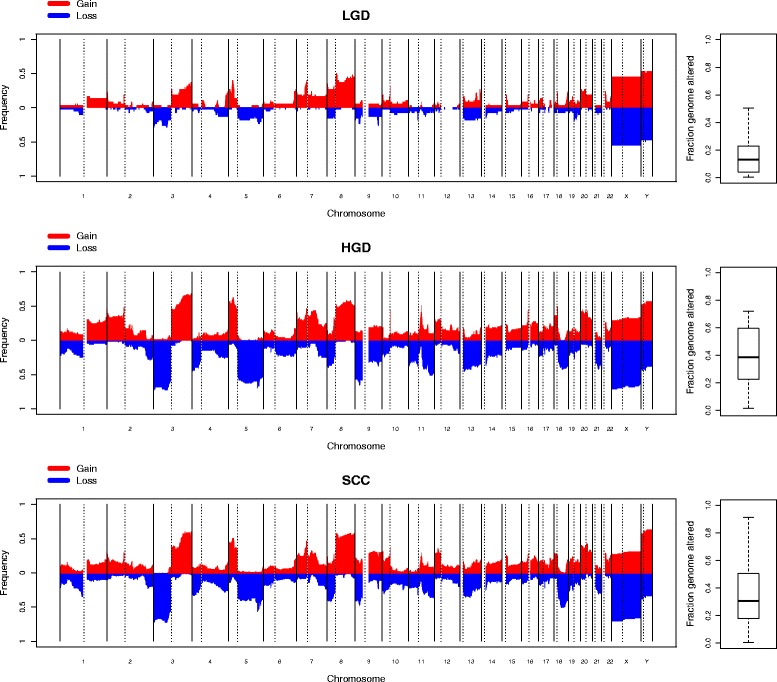
Cumulative copy number frequencies for LGD, HGD and SCC cohorts. The *x-axis* denotes genomic position, while the *y-axis* denotes the proportion of samples with a gain (*red*) or loss (*blue*) at that position. A summary of the fraction genome altered for each sample group is displayed on the *right*

The various LGD, HGD and SCC (*n* = 38, 59 and 149, respectively) samples were examined individually and cumulatively. While there was considerable variation between samples, individually, LGD samples were more likely to have fewer genomic changes than HGD or SCC samples (median fractions genome altered were 0.12, 0.38 and 0.30, respectively). A Mann–Whitney test comparing the distributions of fractions of genome altered between LGD and HGD samples gave a *p* value of 4.76 × 10^−9^. Comparing LGD and SCC samples gave a *p* value of 2.57 × 10^−7^, while comparing HGD and SCC only gave a *p* value of 0.057. This was confirmed by cumulative frequency plots (Fig. 1). The HGD and SCC groups were almost indistinguishable and matched the expected frequencies previously observed in TCGA HNSCC series. The mean copy number profiles of the two groups had a Pearson’s correlation coefficient of 0.92. The correlations between the mean LGD copy number profile and the mean HGD and SCC profiles (both 0.81) were lower than the HGD/SCC comparison. Every region of common gain or loss in one group was reflected in the others. Only the relative frequencies of some of them varied slightly. When the respective frequencies of the most common regions of change in the three sample groups were compared (Table 1) most regions followed the expected pattern, predicted by the overall fraction of genome altered and sample numbers. Gain of chromosome 7 occurred in more LGD samples than would be expected, but this was not significant after adjusting for multiple testing. Only gain of chromosome arm 8q showed a significant deviation, again being present in more LGD samples than expected, indicating that, where present, it is a particularly early event.

**Table 1:** Percentage frequencies of the most common copy number events in LGD, HGD and SCC samples

	LGD	HGD	SCC	*P* value
3p loss	28.95	72.46	0.72	0.3994
4p loss	13.16	44.93	0.33	0.9453
5q loss	23.68	69.57	0.56	0.9787
9p loss	26.32	65.22	0.50	0.7044
11 pq loss	18.42	50.72	0.36	0.7893
18q loss	7.89	42.03	0.50	0.0646
3q gain	36.84	68.12	0.60	0.2041
5p gain	39.47	62.32	0.51	0.0428
7 pq gain	39.47	47.83	0.50	0.0067
8q gain	50.00	57.97	0.59	0.0009
12p gain	7.89	31.88	0.36	0.2404
Expected	12	38	30	

The *p* values that each event differs from the expected frequencies were calculated from the overall fraction of genome altered and total sample number for each group using a chi-squared test. As 11 regions were tested, *p* values below 0.0045 are considered significant

Comparing patterns to those seen in TCGA data [12], these patients showed similar regions of gain and loss, albeit with a few differences. In the HPV-associated patients, neither amplification of the *E2F1* gene nor loss of *TRAF* were observed. In the HPV-negative patients, loss of *TP53* was not observed. However, local gain of 3q26-3q28 was common, occurring in 23 out of 59 patients as either a partial arm gain or local amplification. Similarly, local amplification of 11q23 was observed in 23 patients.

**Fig. 2:**
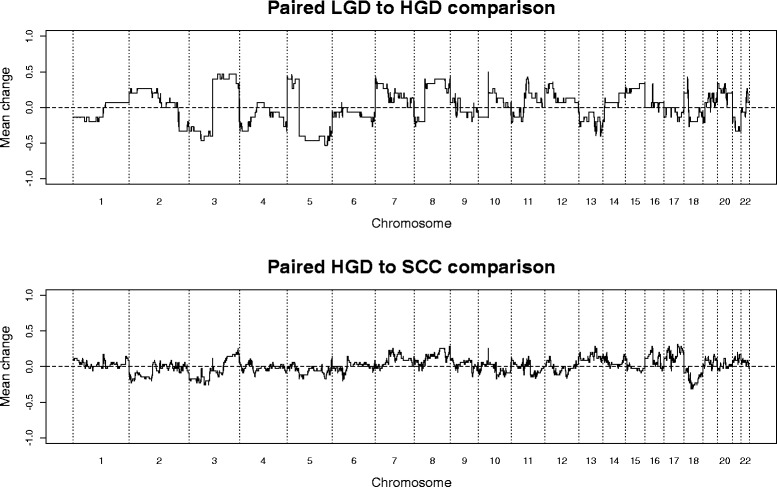
Mean changes in copy number between paired samples. The *top panel* shows the mean of changes seen in HGD samples but not their paired LGD samples. The *bottom panel* shows the mean change seen in SCC samples but not their paired HGD samples

Samples were also examined in the context of other samples from the same patient. For those patients from which LGD and HGD samples were analysed, the mean change across the genome seen in the HGD but not the matching LGD was calculated. A similar analysis was performed with patients with matching SCC and HGD samples (Fig. 2). For the LGD–HGD pairs, the changes seen in HGD samples but not their matched LGD samples very closely mirrored the cumulative frequency plot of SCC samples (correlation coefficient 0.82). In contrast, the changes seen in SCC samples but not the matched HGD samples appeared to be more random, with only a 0.51 correlation coefficient with the overall SCC pattern.

**Fig. 3:**
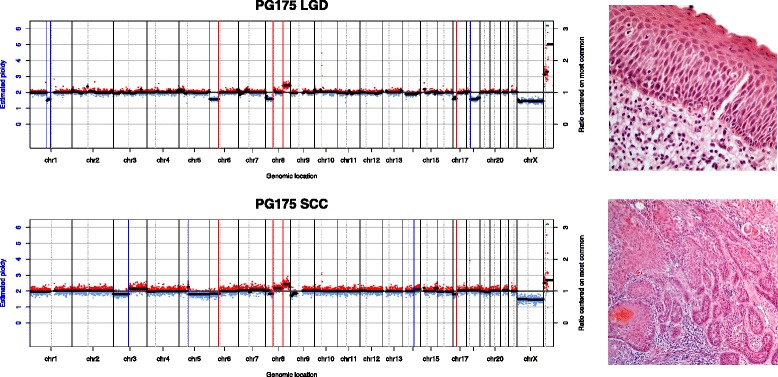
Copy number class 1. Copy number plots from an LGD and SCC sample from the same patient. The *x-axis* shows genomic position and the *y-axis* shows copy number ratio. *Red dots* indicate a ratio above 1 (gain) while *blue dots* indicate a ratio below 1 (loss). Some genomic events (*vertical red lines*) are shared between samples, while some (*blue vertical lines*) are unique to each sample. On the right are high-resolution histology images of the respective samples

**Fig. 4:**
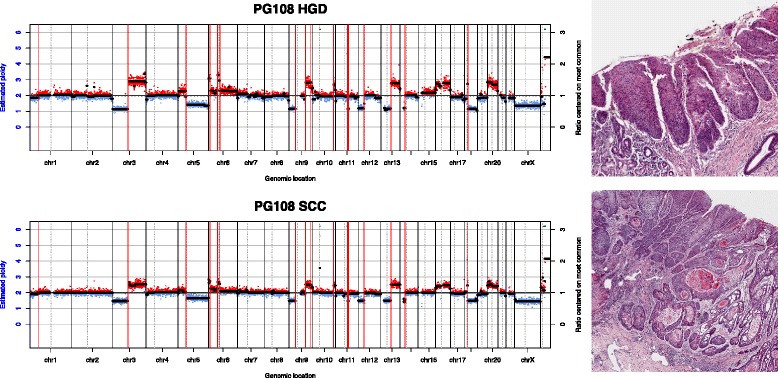
Copy number class 2. Both dysplasia and SCC samples have multiple copy number events, all of which are shared by both samples

**Fig. 5:**
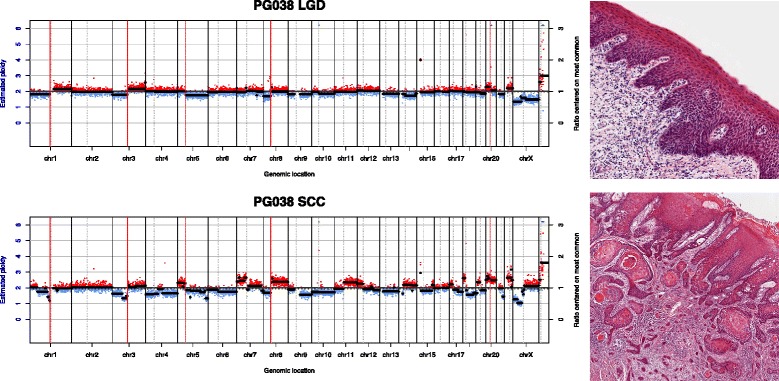
Copy number class 3. All of the events in the dysplasia are also found in the SCC, while the SCC has its own unique events

Patients were subdivided into groups according to the relationships between patterns of the dysplasia and SCC samples: (1) dysplasia and SCC showed some common features, but each displayed other independent features; (2) dysplasia and SCC were nearly identical; (3) the dysplasia showed a subset of the alterations seen in the SCC, or the dysplasia had a flat profile; (4) both dysplasia and SCC had flat profiles. Examples of these relationships are shown in Figs. 3, 4 and 5. Some patients provided more than one sample and could sometimes be classed into more than one of these groups, depending on which samples were compared.

**Fig. 6:**
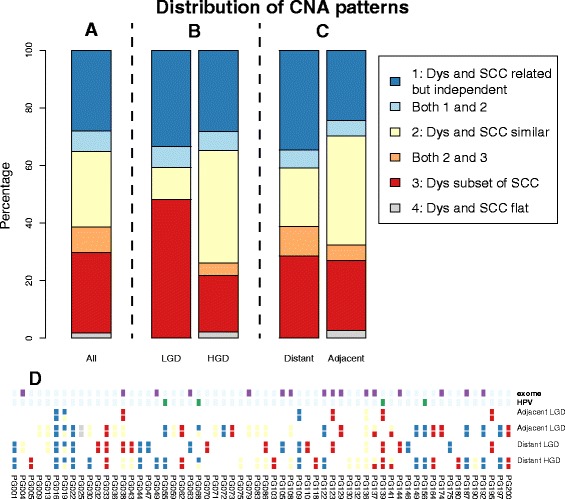
Distribution of copy number alteration (*CNA*) patterns. **a** The pattern for all patients. **b** Comparison of LGD and HGD. **c** Comparison of patients with adjacent and/or distant dysplasia—SCC pairs. **d** Patterns for each individual patient, along with labels for those patients who were either HPV-positive and/or selected for exome sequencing

Percentages of patients in each of these groups are shown in Fig. 6, as well as a patient-by-patient breakdown. Samples are classed according to whether the dysplasias were LGD or HGD and, separately, whether the dysplasias were in the same or a different block to the SCC. Some patients were counted multiple times. For example a patient might have had an adjacent HGD with pattern 1, an adjacent pattern 1 LGD and a distant pattern 2 LGD. This patient would be counted as pattern 1 in the HGD analysis, both 1 and 2 in the LGD analysis, 1 for the adjacent dysplasia analysis and 2 for the distant dysplasia analysis.

Patterns 1, 2 and 3 accounted for all but one patient and were found at very similar overall frequencies. Comparing LGD and HGD samples, both types had very similar frequencies of pattern 1 (related but independent), the LGD samples had relatively higher frequencies of pattern 3 (dysplasia being a simpler version of the SCC), while the HGD samples had a relatively higher proportion of pattern 2 (SCC and dysplasia very similar). A chi-squared test showed this result to be significant (*p* = 0.0264).

When comparing samples with distant and adjacent dysplasia–SCC pairs, the distant samples had a relatively lower frequency of pattern 2 and relatively higher frequencies of patterns 1 and 3. The differences were not as striking as in the LGD–HGD comparison and, consequently, this result was not significant (*p* = 0.6296).

### Exome sequencing

Sixteen patients were selected for exome sequencing of one dysplasia and one SCC sample each. Sequencing statistics for each sample are shown in Additional file 1: Table S2. Between 1146 and 7835 (median 2523) somatic variants were called in either the SCC or dysplasia per patient with initial filters in place. Relatively small numbers of these variants, between 1.9 and 15.9% (median 9.5%) were shared between dysplasia and SCC samples from the same patient. A patient-by-patient breakdown of these results is shown in Additional file 1: Figure S3.

**Fig. 7:**
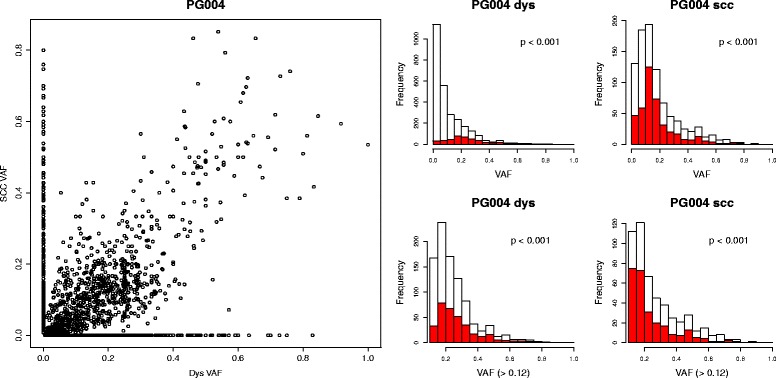
Distribution of shared and private VAFs for patient PG004. In the *left plot*, all putative mutations are displayed as SCC VAF versus dysplasia VAF. Private mutations are on the axes at x = 0 or y = 0. To make the distributions of VAFs more visible, they are displayed as histograms in the *right plots*. On the *left*, the distribution of dysplasia VAFs is shown, with the shared mutations in *red*. On the *right* the SCC VAFs are shown. Many patients had high numbers of putative calls with very low VAFs, which may be spurious and which make the rest of the distribution difficult to see. Therefore, the *bottom two plots* replicate the *top plots*, but only with VAFs >0.12. For each histogram, the *p* value showing the likelihood of the shared and total distributions being the same is shown

When VAFs from paired samples were compared, every patient had a number of private mutations and a diffuse cluster of shared mutations. An example is shown in Fig. 7, with all samples shown in Additional file 1: Figure S6. The private mutations were spread over a range of VAFs, so were not restricted to minor sub-clones. More likely they were mutations which were locally clonal but globally sub-clonal and simply a reflection of sampling. For all 16 patients (16 SCC and 14 dysplasias), the shared mutations had a significantly different distribution of VAFs than the total mutations, with higher values. For eight patients, this was maintained even after VAFs <0.12 were removed. However, the shared VAFs were not confined to the higher end of the range of values. For those samples with a significantly different distribution, the VAFs of shared events still covered the entire range, but with a shift towards higher values. Higher VAFs for shared mutations would indicate that these represented earlier, more common sub-clones, or the result of a selective sweep, with the remaining, low VAFs events being private mutations which occurred since then. The observed similar, or overlapping, distributions are more indicative of spatial sampling, with a mixture of sub-clones being spread throughout the lesion with locally variable population densities.

To ensure that these shared mutations were not simply caused by recurrent FFPE artefacts which could occur in any sample, shared mutation calls between and within patients were compared. For every sample, the number of mutation calls shared with every other sample was collected, both from the same patient and from different patients. Comparing numbers of shared calls between patients establishes a baseline level of how many shared calls are likely FFPE artefacts. These results are summarised in Additional file 1: Figure S5 and show that for every VAF range, the number of shared mutations within a patient was significantly greater than the number of presumably false calls shared between patients. For the calls with a VAF over 0.1, there was no overlap at all between the distributions. The sample pair with the fewest number of shared mutations within a patient had more shared calls than the pair with the most between-patient calls.

No patient showed evidence of more than one cluster of mutations, which might reflect distinct sub-clones which were rare in one sample and more common in the pair, as has been seen in leukaemia [32], and might be a result of a new, selectively advantaged sub-clone emerging.

**Fig. 8:**
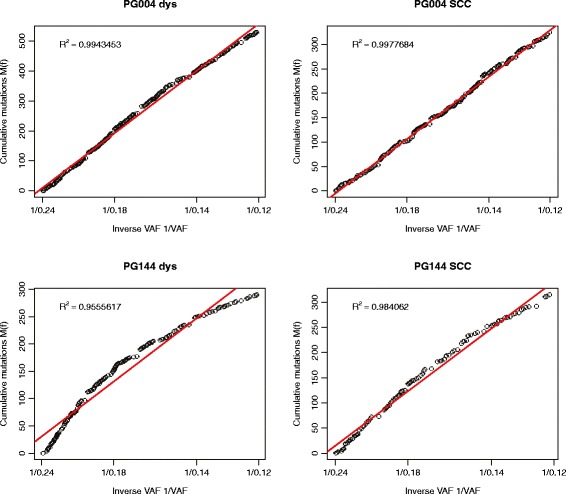
Cumulative distribution of sub-clonal allele frequencies (VAF 0.12–0.24). Neutral evolution is indicated by a straight line with R^2^ values over 0.98. The dysplasia (*left*) and SCC (*right*) from examples of putative neutral (PG004, *top*) and non-neutral (PG144, *bottom*) evolution are shown

We tested for this putative absence of sub-clonal selection by plotting the rate at which sub-clonal mutations emerged. Neutral evolution will see a steady accumulation of mutations. If a new sub-clone causes a selective advantage, then the measured number of mutations per cell division will drop, as the new sub-clone begins to dominate its surroundings, reducing heterogeneity. All 16 SCC samples and 14 of the 16 dysplasias had an R^2^ value over 0.98, which has been calculated to be a stringent indicator of neutrality [6]. Examples of neutral and non-neutral samples are in Fig. 8, with all samples shown in Additional file 1: Figure S7. Both the non-neutral samples showed a reduction of the mutations per cell division over time, indicative of a selectively advantaged sub-clone emerging at an early stage. The lack of this curve in the matching SCC suggests that this clone is fixed in the population by the time the disease becomes invasive, and that further sub-clonal evolution confers no further selective advantage to growth rate. The model uses an “effective cell division rate”, which is division minus cell death. It is not clear if these results show the true emergence of a sub-clone with a selective growth advantage over its neighbours, or whether the overall growth of the lesion increased as the cells moved towards a more invasive phenotype, free of the control mechanisms of normal tissue.

**Fig. 9:**
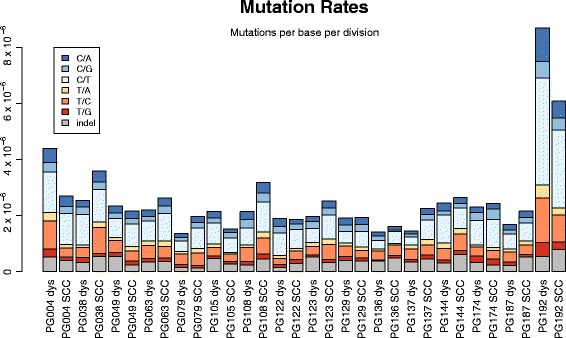
Mutation rates of all samples, calculated as the gradients of the cumulative distribution plots divided by the size of the exome. Mutation types are differentiated by colour

These results also allow for estimations of mutation rate which are less skewed by sequence errors and FFPE artefacts than mere counting of variants, using the gradient of the graphs in the region between the low VAFs, which may be artefacts, and the high VAFs, which may be clonal. While there was variation between samples (Fig. 9), all rates were in line with those taken from TCGA lung series, as calculated by Williams et al. [6]. One sample (PG192) was a clear outlier, with mutation rates more than twice that of the next highest patient. Five patients had a higher rate for dysplasia, 11 for SCC. Again, it should be noted that these rates are mutations per base per effective cell division. A putatively high rate may actually be a normal rate with low cell division—a slow growing disease.

Since we observed that most samples demonstrated essentially neutral evolution, with sub-clonal mutations having little effect on tumour growth, it appeared that potential driver events were more likely to be early, clonal mutations. Therefore, variants were filtered to remove those present in less than 50% of cells in both dysplasia and SCC samples, and those which would have no predicted effect on the protein expressed. The results of this more stringent filtering are shown in Additional file 1: Figure S4. Between 44 and 179 variants per patient passed the filters (median 97). These mutations, with their predicted effects, are listed in full in Additional file 2. The filtered variants were more likely to be shared between dysplasia and SCC samples than the unfiltered variants (*p* < 0.0001). One patient shared 2.7% variants while the rest shared between 11.8 and 53.3% (median 34.3%). All patients had fewer mutations in dysplasia than SCC samples. All but two had more shared mutations than dysplasia-only changes. All but one patient had more SCC-only mutations than shared. Neither the number nor proportion of shared mutations correlated with the total number of mutations per sample. Mutations are listed in Additional file 1: Table S3.

**Fig. 10:**
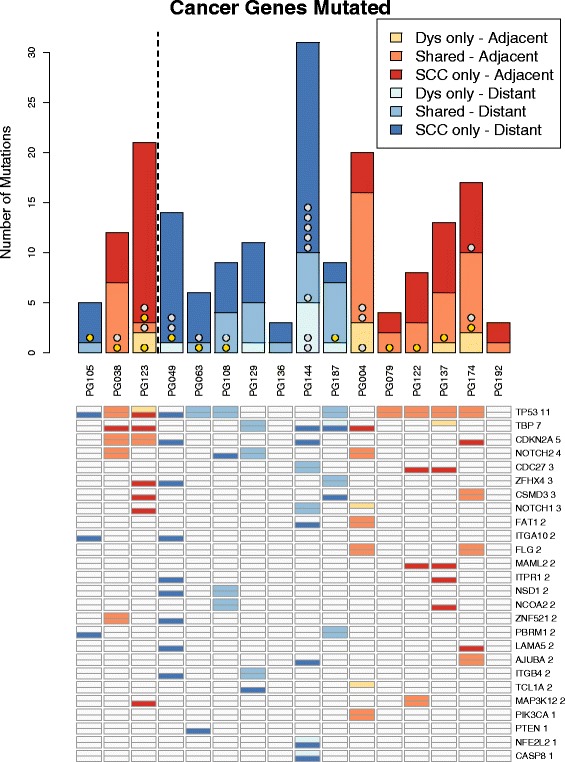
The variants seen affecting putative cancer pathways or lists of common HNSCC genes. *TP53* mutations are shown in *gold*. Other common TCGA mutations are shown in *silver*. Any putative cancer gene mutated in more than one patient plus all common TCGA mutations are listed. *Shades of orange* indicate adjacent dysplasia–SCC pairs. *Shades of blue* indicate distant pairs. For each patient, the number of dyplasia-only, shared and SCC-only variants are shown. There are two rows for each gene; the *top row* indicates that it is mutated in dysplasia and the *bottom row* in SCC. So for *TP53*, for example, PG105 has a SCC-only mutation, PG038 has a mutation which is shared, and PG123 has different mutations in the dysplasia and SCC samples. All genes are shown in Additional file 1: Figure S8

To further investigate putative driver genes, the variants were filtered against lists of genes in pathways predicted to be altered in cancer, as well as lists of genes observed to be frequently mutated in HNSCC. These results are shown in Fig. 10, Additional file 1: Figure S8 and Additional file 1: Table S3. The numbers of genes affected varied considerably between patients—between 3 and 31 (median 10). Between 0 and 67% of these were shared between samples. The relationships between the number of SCC-only, shared and dysplasia-only mutations was not as straightforward has had been seen in the numbers of filtered mutations. Only eight patients had any dysplasia-only changes in suspected cancer genes. All patients had some SCC only changes. One had no shared events. Four patients had more shared than SCC mutations, 11 had more SCC than shared, while one had equal numbers of shared and SCC-only changes. No pattern could be observed linking these numbers to either dysplasia grade or whether the dysplasia was adjacent or distant to the SCC. Similarly, the proportion of shared variants was not significantly different to the initial list of filtered variants (*p* = 0.921). If these were all driver genes, then it would be expected that they would be more likely to be early, shared events than the list of filtered genes. As this was not the case, it seems that many of the genes on these list were merely potential drivers, rather than actively driving these lesions.

Particular attention was paid to genes present in over 10% of samples in TCGA HNSCC survey. *TP53* was mutated in 11 of the 16 patients. For eight of these patients, the *TP53* mutation was shared between the SCC and dysplasia, indicating that it was an early event. Two patients had mutated *TP53* in their SCC but not their dysplasia samples (both of these dysplasia samples were in a different FFPE block to the SCC). One patient had separate *TP53* mutations in both the dysplasia and the SCC, which were not shared between the two. Other TCGA genes were not shared as frequently: seven genes shared, ten SCC-only and three dysplasia-only. Interestingly, one of the samples with no *TP53* mutations had the highest numbers of other common TCGA genes mutated.

Finally, the filtered variants were used to generate lists of Gene Ontology (GO) terms significantly affected by the dysplasia-only, shared and SCC-only mutations from each patient. These results are shown in Additional file 1: Table S4. For 11 of the 16 patients, no dysplasia-only GO terms were significantly enriched. Every patient had some GO terms enriched that could realistically be linked to carcinogenesis, such as apoptosis, cell adhesion, cell differentiation, response to wounding, etc. However, there was little overlap between patients, or any particular pattern of some terms appearing in the shared lists and some in the SCC-only lists.

## Discussion

The frequent close physical association between oral dysplasia and invasive carcinoma has led to the common assumption that the relationship between the two is sequential, with one arising from the other [33–36]. A common approach is to observe groups of either dysplasias or carcinomas and compare them to each other, with some evidence suggesting that lower grade dysplasias have lower risk of progression [7, 8]. Recent comprehensive characterisation of fully invasive disease has suggested that either *TP53* mutation or HPV infection are almost universal, along with a small number of common copy number patterns, but that changes to other genes were far more heterogeneous, with only six other genes mutated in over 10% of samples, and none of those in more than 25% [12].

The purpose of this study was to investigate the genomic similarities and differences between LGD, HGD and SCC. As well as studying groups of samples of similar grade, we were able to observe the changes in individual patients, which is important given how few common changes there are in HNSCC. Was it the case that certain groups of changes always occurred in a specific order, and how similar are the dysplasia and carcinoma samples from each patient? Additionally, this work also allowed us to test the hypothesis that dysplasia is a simpler precursor to invasive disease. Seminal work in colorectal cancer has suggested a simple step-wise genomic development from normal tissue, through pre-cancerous stages of increasing grade to fully invasive disease [2]. In previous work we have examined clonal relationships between dysplasia and carcinoma in five patients, which suggested that the relationships are sometimes complex and are not always a simple progression [15]. By working with larger numbers of patients, we were able in this study to examine trends between the different grades of disease.

By examining the frequencies of copy number gains and losses in the cohorts of LGD, HGD and SCC samples, genomic damage did appear to be linked to grade. HGD samples showed copy number patterns almost indistinguishable from those of SCC samples. Only a minority of LGD samples displayed these changes, with many changes in HGD and SCC samples absent. These findings suggest that these copy number changes were not necessary to develop LGD, and tended to occur at some point in the development from LGD to HGD. The HGD samples contained all the common copy number changes seen in the invasive disease, so the final trigger for invasion was more likely to be a gene mutation or some other transcriptomic, epigenetic or environmental change. Forty percent of dysplasia samples displayed independent evolution of the genome absent in the SCC, showing that the samples taken were not the direct progenitors of the invasive disease.

A similar pattern was observed when examining the shared and private mutations seen in the exome data. The shared mutations tended (but not overwhelmingly) to have higher VAFs, indicative of early sub-clones. The presence of large numbers of low VAF shared mutations in all samples does suggest that the same minor sub-clones are present in both the dysplastic and invasive part of a lesion, and that mutationally similar cells may appear histologically different, perhaps depending on the status of the majority of their neighbours. If, instead, the invasive process had begun with a single sub-clone in the dysplasia, which proceeded without mixing with any of its neighbours, then all the shared mutations would have a high VAF in the SCC sample.

We examined this gradual model of tumour evolution by testing whether mutations were accumulating according to a neutral or selective pattern. Two dysplasia samples showed evidence of early selection, but the remaining dysplasias and all SCCs followed the neutral predictions. Neutral evolution was even found in patients such as PG049, where all the known driver mutations occurred only in the SCC sample, which might suggest a late occurring selective advantage. This could be due to the fact that only one dysplasia sample was taken for each patient. More extensive sampling might have detected these mutations in a region of the dysplasia more directly ancestral to the SCC, as we sometimes found when we sampled multiple regions of a smaller group of patients [15]. An alternative explanation is that these driver mutations only affected the invasive potential of the lesions and had less effect on their growth rate. Even though we only sequenced 16 patients, no other cancer type examined by Williams et al. [6] displayed neutral evolution in more than half of the patients examined. If we were expecting 50% of samples to be neutral, observing neutrality in 14 has a *p* value of 0.0027. This unusually high frequency could be explained by the natural history of HNSCC. It is typically caused by decades of tobacco use, with attendant constant gradual mutation in the oral epithelium. However, in contrast with lung cancer, oral cancer is in one of the most sensitive and accessible regions of the body, so is frequently diagnosed extremely early, possibly before the emergence of later, selectively advantaged populations.

Since no samples showed evidence of a late-emerging driver clone, we concentrated on mutations in a high proportion of cells (in either dysplasia or SCC) which might have a putative effect on protein function.

As with the copy number analysis, once samples within patients were compared, the variability between patients was stark, in both numbers of overall mutations and the proportions of those mutations which were dysplasia-only, shared, or SCC-only.

Known cancer genes were very infrequently mutated only in the dysplasia samples, suggesting that whilst dysplasia may sometimes evolve away from the recent common ancestor, much of this evolution consists of the accumulation of passenger events. Cancer gene mutations were equally likely to be shared, or SCC-only, depending on the patient. *TP53* mutations, however, were almost always shared, indicating that, where present, they were necessary, early events in the development of HNSCC.

When examining GO terms, very few terms were enriched from the dysplasia-only lists. This confirmed the hypothesis that the many mutations accumulating as dysplasia and SCC evolve away from the common ancestor are sporadic passengers. Once mutations that perturb a pathway begin to accumulate, this fraction of cells is more likely to be represented in the invasive disease. Which pathway is perturbed does not appear to be shared between patients.

## Conclusions

Taken together, these results present a fascinating insight into the early stages of HNSCC development. Copy number changes and point mutations do appear to accumulate as the disease moves from normal tissue to LGD, HGD and SCC, but there does not appear to be a step-wise appearance of a wave of sub-clones, each replacing its ancestor in a selective sweep. The finding of multiple low VAF shared mutations raises the intriguing possibility that the behaviour of cells may be influenced by their neighbours, as has been observed in other cancer types [37–40]. We do see an increase in mutations and copy number changes as we move from LGD to HGD to SCC, but this does not appear to correspond to a matching change in selective advantage. It is speculation at this stage, but it could be that new sub-clones do not increase in relative frequency in a lesion because they create an environment in which their sister sub-clones can also flourish. Similar to the concept of field cancerisation [36], the long period of tobacco exposure in most patients may produce a sub-clinical population of cells which are primed for disease, and once a suitable combination of mutations emerges, not just the cells directly mutated change their phenotype.

Our findings could have significance for future efforts to monitor patients with dysplasia, when deciding whether or not to intervene. Although dysplasia samples did have mutations not found in the associated SCC, these were not usually in known cancer genes. Mutations in cancer genes in the dysplasia samples were mostly also found in the associated SCC samples, so could be used as markers of progression.

## Additional files

Additional file 1: An example of tissue selection, a full sample list, exome sequencing metrics, full VAF and neutrality comparisons, lists of genes mutated, and lists of GO terms enriched. (PDF 8250 kb)

Additional file 2: For each patient undergoing exome sequencing, a full list of the mutations passing filters and their predicted effects are given in excel files. These files are the Variant Effect Predictor output for the genes present in over 50% of cells in either dysplasia or SCC, which are dysplasia-only, shared, or SCC-only. Note that due to multiple possible transcripts, most mutations are listed multiple times, with multiple possible effects. (ZIP 2331 kb)
