# Supplementary material for: The genomic road to invasion—examining the similarities and differences in the genomes of associated oral pre-cancer and cancer samples
Source: Genome Med. 2017 Jun 7;9:53. doi: 10.1186/s13073-017-0442-0 (PMC5461742; doi:10.1186/s13073-017-0442-0)
Supplement: Supplementary file 1 — An example of tissue selection, a full sample list, exome sequencing metrics, full VAF and neutrality comparisons, lists of genes mutated, and lists of GO terms enriched. (PDF 8250 kb) [file 13073_2017_442_MOESM1_ESM.pdf]

Supplementary information for Wood *et al*: The genomic road to invasion – examining the similarities and differences in the genomes of associated oral pre-cancer and cancer samples

Figure S1 - Tissue sections of PG038 to illustrate regions chosen for micro-dissection. Stained sections were examined by microscopy and the regions of interest were marked by one of the study pathologists (top row). The bottom row shows the highlighted regions at higher magnification, indicating normal tissue, low grade dysplasia and invasive carcinoma.

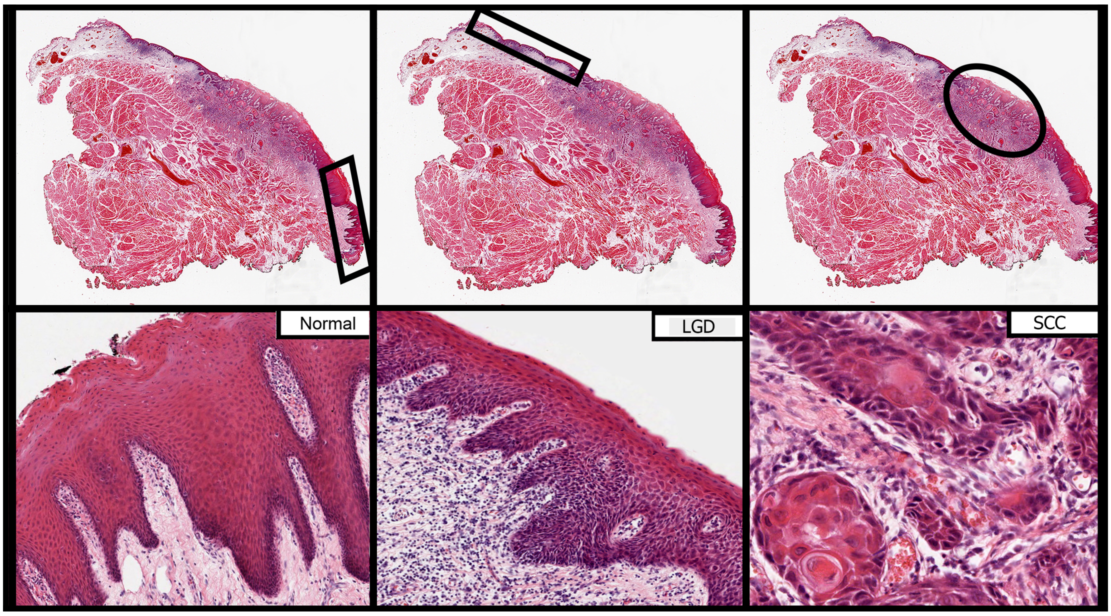

Table S1 – Details of all samples used for CNA analysis. Displayed are patient ID, unique sample ID, sample grade (LGD, HGD or SCC) and whether any other samples from the same patient were taken from the same block.

patient	sample_ID	histology	adjacent
PG001	1TZ	LGD	none
PG001	1U3	LGD	1U1
PG001	1U1	HGD	1U3
PG001	1U5	SCC	none
PG002	1U7	HGD	none
PG002	1U9	HGD	none
PG003	1UB	LGD	1UD
PG003	1UD	HGD	1UB
PG004	1UX	LGD	1UV
PG004	1UV	LGD	1UX
PG004	1UP	SCC	none
PG004	1UT	SCC	none
PG004	1UZ	SCC	none
PG004	1V3	SCC	none
PG005	349	HGD	none
PG006	34R	LGD	none
PG009	34S	HGD	34T
PG009	34T	SCC	34S
PG011	1VH	HGD	none
PG011	1VJ	HGD	none
PG013	1VM	LGD	none
PG013	34U	HGD	1VL
PG013	1VL	SCC	34U
PG016	34V	LGD	none
PG016	34W	LGD	2DF,34X
PG016	34X	HGD	2DF,34W
PG016	13C	SCC	none
PG016	2DF	SCC	34W,34X
PG019	2ZB	HGD	2ZC,08N
PG019	2ZC	SCC	08N,2ZB
PG019	08N	SCC	2ZC,2ZB
PG019	2ZE	SCC	none
PG019	099	SCC	none
PG019	097	SCC	none
PG019	2ZF	SCC	none
PG019	08H	HGD	08J
PG019	08J	SCC	08H
PG019	13T	SCC	none
PG019	08S	SCC	none
PG019	08V	SCC	none

PG019	08X	SCC	2YE
PG019	2YE	SCC	08X
PG019	08K	HGD	094
PG019	094	HGD	08K
PG022	3DZ	LGD	none
PG022	13X	LGD	none
PG022	13W	LGD	none
PG022	3DV	HGD	none
PG022	3DX	HGD	3DY
PG022	3DR	HGD	none
PG022	13V	HGD	none
PG022	3DT	SCC	none
PG022	3DU	SCC	none
PG022	3DW	SCC	none
PG022	3DY	SCC	3DX
PG024	2DS	LGD	none
PG025	33S	HGD	3BX
PG025	3BX	SCC	33S
PG030	2ES	SCC	none
PG030	07P	SCC	none
PG030	07Q	SCC	none
PG030	07S	SCC	none
PG030	09A	SCC	none
PG030	07Y	SCC	none
PG030	227	SCC	none
PG030	22C	SCC	none
PG032	2EX	LGD	none
PG032	2DT	SCC	none
PG032	2DV	SCC	none
PG033	2S1	LGD	none
PG033	2RX	HGD	none
PG033	2RZ	HGD	2S0
PG033	2RY	SCC	none
PG033	2S0	SCC	2RZ
PG033	2S4	SCC	none
PG033	35N	SCC	35P
PG033	35P	SCC	35N
PG036	351	HGD	2DW,350
PG036	2DW	SCC	350,351
PG036	350	SCC	2DW,351
PG038	2DZ	LGD	2E0
PG038	2DX	HGD	2DY
PG038	2DY	SCC	2DX
PG038	2E0	SCC	2DZ
PG043	2SB	LGD	none
PG043	2SA	HGD	none

PG043	2S9	SCC	none
PG044	34D	LGD	34E
PG044	34E	LGD	34D
PG044	34C	HGD	none
PG044	2E1	SCC	354
PG044	354	SCC	2E1
PG047	2SZ	LGD	none
PG047	2SY	SCC	none
PG047	2T0	SCC	none
PG055	080	SCC	none
PG055	083	SCC	none
PG055	085	SCC	none
PG055	14G	SCC	none
PG056	355	LGD	34F,34G
PG056	34F	HGD	34G,355
PG056	34G	HGD	34F,355
PG059	357	HGD	2E4
PG059	2E4	SCC	357
PG062	2TC	HGD	2TD
PG062	2TA	SCC	2TB
PG062	2TB	SCC	2TA
PG062	2TD	SCC	2TC
PG063	359	LGD	none
PG063	2E6	SCC	none
PG068	2TH	HGD	2TJ
PG068	2T6	SCC	2T7
PG068	2T7	SCC	2T6
PG068	2TJ	SCC	2TH
PG070	2TN	LGD	none
PG070	2TK	SCC	none
PG070	2TM	SCC	none
PG071	2E7	HGD	2E8
PG071	2E8	SCC	2E7
PG072	3BU	HGD	2TP
PG072	2TP	SCC	3BU
PG072	2TT	SCC	none
PG073	33V	SCC	none
PG075	3C0	HGD	none
PG075	2TX	SCC	none
PG075	2TZ	SCC	none
PG079	3Q2	HGD	3Q3
PG079	3Q3	SCC	3Q2
PG079	2EA	SCC	none
PG083	2EL	HGD	2EM
PG083	2F8	HGD	2F7
PG083	0D1	SCC	none

PG083	2EM	SCC	2EL
PG083	2F4	SCC	none
PG083	2F6	SCC	none
PG083	2F7	SCC	2F8
PG083	2F3	SCC	none
PG085	32Z	HGD	none
PG086	2SG	LGD	none
PG086	2SD	HGD	2SE
PG086	2SE	SCC	2SD
PG086	2SF	SCC	none
PG086	14M	SCC	none
PG103	332	SCC	333
PG103	333	SCC	332
PG103	336	SCC	none
PG103	338	SCC	none
PG105	2FK	LGD	none
PG105	2FE	HGD	2FF
PG105	2FH	HGD	2FJ
PG105	14P	SCC	none
PG105	2FF	SCC	2FE
PG105	2FJ	SCC	2FH
PG108	2FL	HGD	none
PG108	2FM	HGD	2FN
PG108	2FN	SCC	2FM
PG109	2FQ	LGD	2FR
PG109	2FR	LGD	2FQ
PG109	2FS	LGD	2FT
PG109	2FU	HGD	none
PG109	2FT	SCC	2FS
PG110	33B	LGD	none
PG110	339	SCC	33A
PG110	33A	SCC	339
PG118	2FZ	SCC	none
PG122	3PY	HGD	3PZ
PG122	3PZ	SCC	3PY
PG122	2G8	SCC	none
PG123	3PU	LGD	2KB,3PW
PG123	3PW	SCC	2KB,3PU
PG123	2K9	SCC	2KA
PG123	2KA	SCC	2K9
PG123	2KB	SCC	3PU,3PW
PG129	2KD	HGD	2KE,2KF
PG129	2KE	HGD	2KD,2KF
PG129	2KF	SCC	2KD,2KE
PG130	2KJ	HGD	2KK
PG130	2KK	HGD	2KJ

PG130	2KM	HGD	none
PG130	2KN	SCC	none
PG132	344	HGD	none
PG132	343	SCC	none
PG132	345	SCC	none
PG132	347	SCC	none
PG136	086	SCC	none
PG136	08B	SCC	none
PG137	2L4	LGD	none
PG137	3Q0	HGD	2L3,3Q1
PG137	3Q1	SCC	2L3,3Q0
PG137	2L1	SCC	none
PG137	2L3	SCC	3Q0,3Q1
PG137	2L5	SCC	none
PG137	0D7	SCC	none
PG139	2LD	LGD	2LC
PG139	2LF	HGD	none
PG139	2L8	SCC	2L9,35F
PG139	2L9	SCC	2L8,35F
PG139	2LC	SCC	2LD
PG139	2LG	SCC	none
PG139	35F	SCC	2L8,2L9
PG141	35G	HGD	0DF,2LJ
PG141	35J	HGD	2LK,35H
PG141	0DF	SCC	2LJ,35G
PG141	2LJ	SCC	0DF,35G
PG141	2LK	SCC	35H,35J
PG141	2LM	SCC	none
PG141	35H	SCC	2LK,35J
PG144	2LU	LGD	none
PG144	2LT	SCC	none
PG146	2LX	LGD	none
PG146	2LV	SCC	none
PG146	2LY	SCC	none
PG149	2LZ	HGD	none
PG149	2M0	HGD	1F6,2M1
PG149	1F6	SCC	2M0,2M1
PG149	2M1	SCC	1F6,2M0
PG150	2M2	LGD	none
PG156	2M7	HGD	2M8
PG156	2M8	SCC	2M7
PG156	2M9	SCC	2MA
PG156	2MA	SCC	2M9
PG164	2ST	HGD	2SS
PG164	2SQ	SCC	none
PG164	2SR	SCC	35S,35T,35U

PG164	2SS	SCC	2ST
PG164	35T	SCC	2SR,35S,35U
PG164	35U	SCC	2SR,35S,35T
PG164	35S	SCC	2SR,35T,35U
PG175	2MT	LGD	none
PG175	2MP	SCC	none
PG180	2MU	SCC	none
PG187	2MZ	HGD	none
PG187	34L	HGD	34M,3EL
PG187	34M	HGD	34L,3EL
PG187	3EK	HGD	none
PG187	3EL	SCC	34L,34M
PG187	2N0	SCC	none
PG188	2N1	HGD	2N2,2N3
PG188	2N2	HGD	2N1,2N3
PG188	2N3	HGD	2N1,2N2
PG190	2R8	HGD	none
PG190	2R9	HGD	none
PG190	2R7	SCC	none
PG192	2RB	HGD	2RC,2RD
PG192	2RC	HGD	2RB,2RD
PG192	2RD	SCC	2RB,2RC
PG192	2RE	SCC	none
PG195	2RH	LGD	2RJ
PG195	2RG	SCC	none
PG195	2RJ	SCC	2RH
PG195	2RK	SCC	none
PG196	2RM	LGD	none
PG197	2RP	HGD	none
PG197	2RQ	SCC	none
PG199	2RU	HGD	none
PG199	35L	HGD	none
PG200	2RV	HGD	none
PG200	2RW	SCC	none

Table S2 – Exome sequencing statistics. Displayed for each sample, are the number of reads, aligned reads, bases sequenced, median on-target coverage, the number of mismatches (total mismatches, not just called mutations), the number of mismatches per base, the number of C>T (or G>A) mismatches, the C>T (G>A) mismatch rate per base, and per mismatch. The number of mismatches and proportion of those which are C>T(G>A) are known to be a way of measuring damage by formalin fixation.

Name	Reads	Aligned	Bases	Median coverage	Mismatches	Mismatches per base	C>T	C>T per base	C>T per mismatch
PG004-blood	3.89E+07	3.83E+07	3.05E+09	91	3.68E+06	1.21E-03	1.13E+06	3.71E-04	0.31
PG004-dys	6.29E+07	5.81E+07	4.38E+09	95	1.17E+07	2.68E-03	4.34E+06	9.92E-04	0.37
PG004-SCC	8.78E+07	8.61E+07	6.72E+09	97	9.69E+06	1.44E-03	3.47E+06	5.16E-04	0.36
PG038-blood	7.87E+07	7.79E+07	6.43E+09	166	7.69E+06	1.20E-03	2.21E+06	3.45E-04	0.29
PG038-dys	1.05E+08	9.85E+07	7.28E+09	171	1.52E+07	2.08E-03	5.29E+06	7.26E-04	0.35
PG038-SCC	9.54E+07	9.33E+07	7.09E+09	169	9.02E+06	1.27E-03	3.16E+06	4.45E-04	0.35
PG049-blood	8.27E+07	8.19E+07	6.58E+09	177	7.78E+06	1.18E-03	2.38E+06	3.62E-04	0.31
PG049-dys	7.87E+07	7.73E+07	5.93E+09	147	1.09E+07	1.84E-03	3.93E+06	6.63E-04	0.36
PG049-SCC	9.71E+07	9.56E+07	7.39E+09	148	8.79E+06	1.19E-03	3.09E+06	4.18E-04	0.35
PG063-blood	6.36E+07	6.29E+07	5.02E+09	115	5.81E+06	1.16E-03	1.79E+06	3.57E-04	0.31
PG063-dys	9.86E+07	9.73E+07	7.40E+09	132	8.78E+06	1.19E-03	3.16E+06	4.27E-04	0.36
PG063-SCC	1.10E+08	1.08E+08	8.54E+09	132	1.04E+07	1.22E-03	3.69E+06	4.32E-04	0.35
PG079-blood	4.82E+07	4.76E+07	3.77E+09	86	4.42E+06	1.17E-03	1.34E+06	3.54E-04	0.30
PG079-dys	9.57E+07	9.32E+07	6.99E+09	107	1.05E+07	1.50E-03	3.92E+06	5.61E-04	0.37
PG079-SCC	1.05E+08	1.03E+08	7.75E+09	109	1.07E+07	1.38E-03	3.98E+06	5.14E-04	0.37
PG105-blood	8.55E+07	8.46E+07	6.98E+09	179	8.51E+06	1.22E-03	2.42E+06	3.46E-04	0.28
PG105-dys	8.45E+07	8.20E+07	6.36E+09	159	1.04E+07	1.64E-03	3.65E+06	5.74E-04	0.35
PG105-SCC	8.85E+07	8.72E+07	6.84E+09	160	7.99E+06	1.17E-03	2.67E+06	3.91E-04	0.33
PG108-blood	5.99E+07	5.93E+07	4.71E+09	133	5.48E+06	1.16E-03	1.67E+06	3.55E-04	0.31
PG108-dys	8.71E+07	8.41E+07	6.34E+09	196	1.07E+07	1.68E-03	3.76E+06	5.92E-04	0.35
PG108-SCC	1.08E+08	1.06E+08	8.08E+09	203	9.82E+06	1.22E-03	3.34E+06	4.14E-04	0.34
PG122-blood	5.32E+07	5.25E+07	4.19E+09	91	4.97E+06	1.19E-03	1.50E+06	3.58E-04	0.30
PG122-dys	9.41E+07	9.26E+07	7.06E+09	116	9.31E+06	1.32E-03	3.30E+06	4.68E-04	0.35
PG122-SCC	8.92E+07	8.79E+07	6.82E+09	118	8.77E+06	1.29E-03	3.05E+06	4.47E-04	0.35
PG123-blood	7.54E+07	7.46E+07	5.89E+09	159	7.42E+06	1.26E-03	2.20E+06	3.74E-04	0.30
PG123-dys	1.06E+08	1.04E+08	8.18E+09	188	1.07E+07	1.31E-03	3.75E+06	4.58E-04	0.35
PG123-SCC	8.26E+07	8.13E+07	6.22E+09	183	7.95E+06	1.28E-03	2.81E+06	4.51E-04	0.35
PG129-blood	6.76E+07	6.69E+07	5.30E+09	124	6.70E+06	1.26E-03	1.99E+06	3.75E-04	0.30
PG129-dys	8.41E+07	8.21E+07	7.40E+09	93	1.31E+07	1.78E-03	4.48E+06	6.06E-04	0.34
PG129-SCC	1.08E+08	1.06E+08	8.84E+09	94	1.19E+07	1.35E-03	3.99E+06	4.52E-04	0.34
PG136-blood	4.44E+07	4.38E+07	3.61E+09	75	4.38E+06	1.21E-03	1.33E+06	3.68E-04	0.30
PG136-dys	8.40E+07	8.27E+07	6.55E+09	112	8.12E+06	1.24E-03	2.79E+06	4.25E-04	0.34
PG136-SCC	7.82E+07	7.71E+07	6.02E+09	112	7.83E+06	1.30E-03	2.60E+06	4.32E-04	0.33
PG137-blood	5.00E+07	4.93E+07	3.97E+09	107	4.81E+06	1.21E-03	1.44E+06	3.61E-04	0.30
PG137-dys	1.05E+08	1.03E+08	7.69E+09	116	1.07E+07	1.39E-03	3.94E+06	5.13E-04	0.37
PG137-SCC	8.61E+07	8.43E+07	6.31E+09	114	8.81E+06	1.40E-03	3.24E+06	5.14E-04	0.37

PG144-blood	6.24E+07	6.16E+07	4.89E+09	140	6.12E+06	1.25E-03	1.85E+06	3.77E-04	0.30
PG144-dys	5.16E+07	4.98E+07	4.42E+09	107	9.07E+06	2.05E-03	3.15E+06	7.13E-04	0.35
PG144-SCC	7.55E+07	7.30E+07	6.58E+09	117	1.01E+07	1.53E-03	3.26E+06	4.95E-04	0.32
PG174-blood	5.00E+07	4.94E+07	4.11E+09	119	4.99E+06	1.22E-03	1.52E+06	3.71E-04	0.31
PG174-dys	3.97E+07	3.84E+07	3.50E+09	37	6.39E+06	1.83E-03	2.25E+06	6.43E-04	0.35
PG174-SCC	1.38E+08	1.36E+08	1.05E+10	44	1.40E+07	1.33E-03	4.69E+06	4.46E-04	0.33
PG187-blood	5.02E+07	4.94E+07	3.93E+09	123	4.69E+06	1.19E-03	1.42E+06	3.60E-04	0.30
PG187-dys	5.53E+07	5.41E+07	4.35E+09	174	5.99E+06	1.38E-03	2.06E+06	4.74E-04	0.34
PG187-SCC	9.82E+07	9.62E+07	7.38E+09	183	1.05E+07	1.42E-03	3.59E+06	4.86E-04	0.34
PG192-blood	3.76E+07	3.71E+07	3.00E+09	97	3.64E+06	1.21E-03	1.09E+06	3.63E-04	0.30
PG192-dys	3.59E+07	3.25E+07	2.36E+09	39	3.48E+06	1.48E-03	1.43E+06	6.08E-04	0.41
PG192-SCC	3.85E+07	3.30E+07	2.37E+09	35	4.60E+06	1.94E-03	1.80E+06	7.59E-04	0.39

Figure S2: - Confirmation of VAFs cutoffs used for exome data. From Wood *et al, J Pathol* 2015, **237:**296-306, 92 putative mutations called from exome data of seven HNSCC FFPE samples were validated using high coverage (>1000X) coverage sequencing of PCR products. Overall 13 false positives were discovered, of which five were from one sample (PG008), which presumably was of lower quality. If the proposed VAF cutoff of 0.12 was applied, overall the false positive rate was 0.056. Discarding the poor quality sample, the false positive rate reduced to 0.02 (1 of 47 calls).

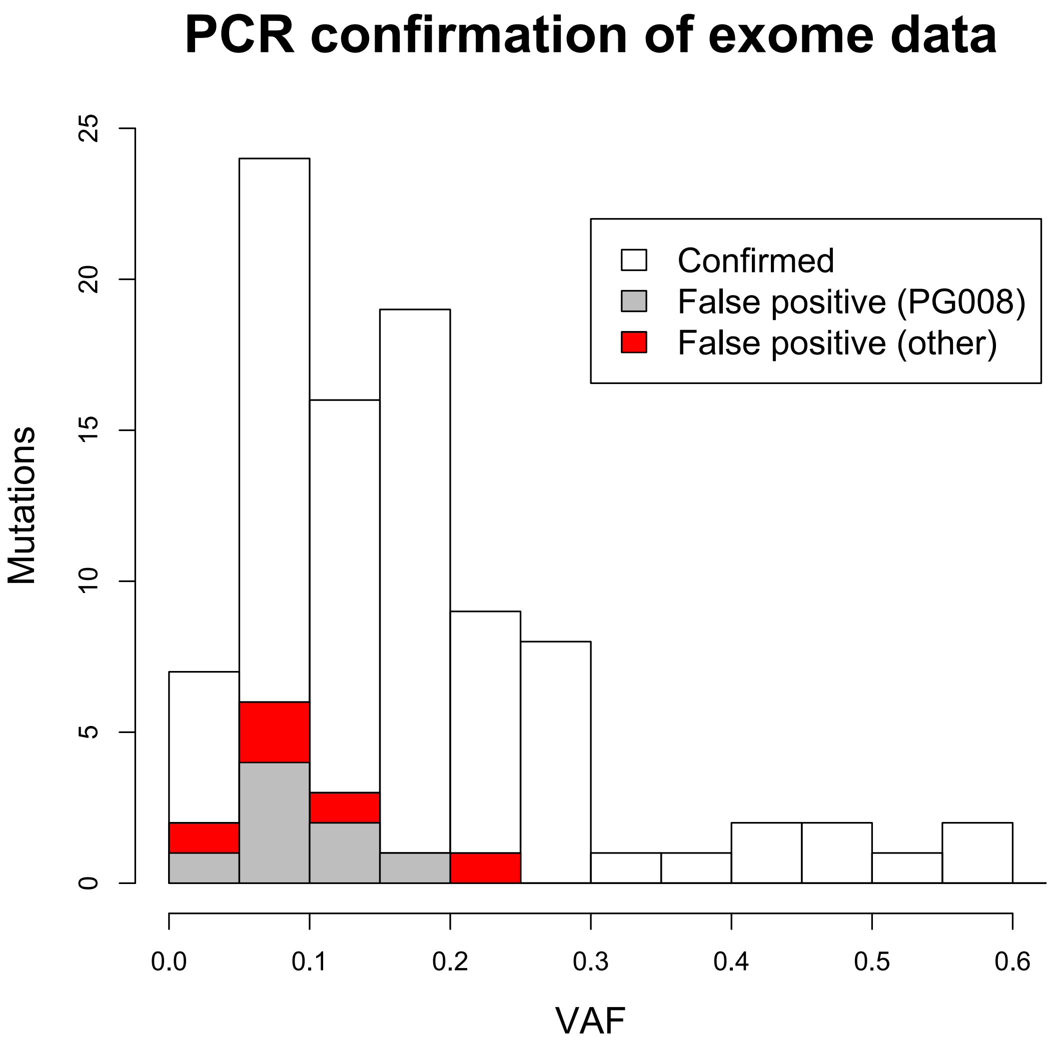

Figure S3: – Numbers of variants with basic filtering. Patients to the left of the dashed line had LGD, to the right HGD. Shades of orange indicate adjacent dysplasia – SCC pairs. Shades of blue indicate distant pairs. For each patient, the number of dyplasia only, shared and SCC only variants are shown.

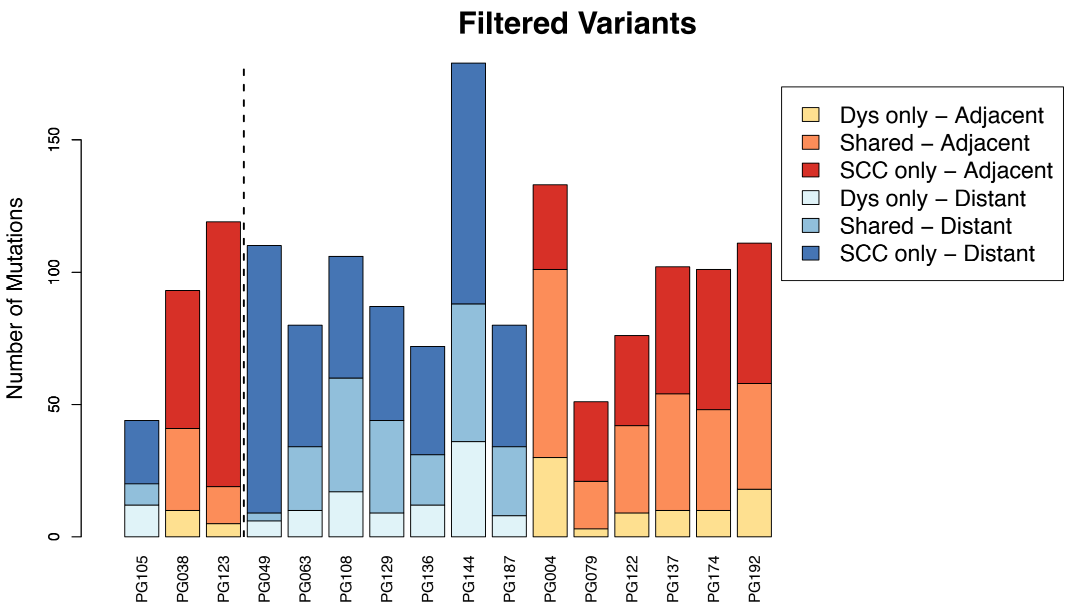

Figure S4 – The numbers of variants after filtering for cellularity and functional effect. Any gene mutated in three or more patients in displayed in the bottom portion of the figure. There are two rows for each gene. The top row indicates that it is mutated in dysplasia and the bottom row SCC. So for *TP53* for example, PG105 has a SCC only mutation, PG038 has a mutation which is shared, and PG123 has different mutations in the dysplasia and SCC sample.

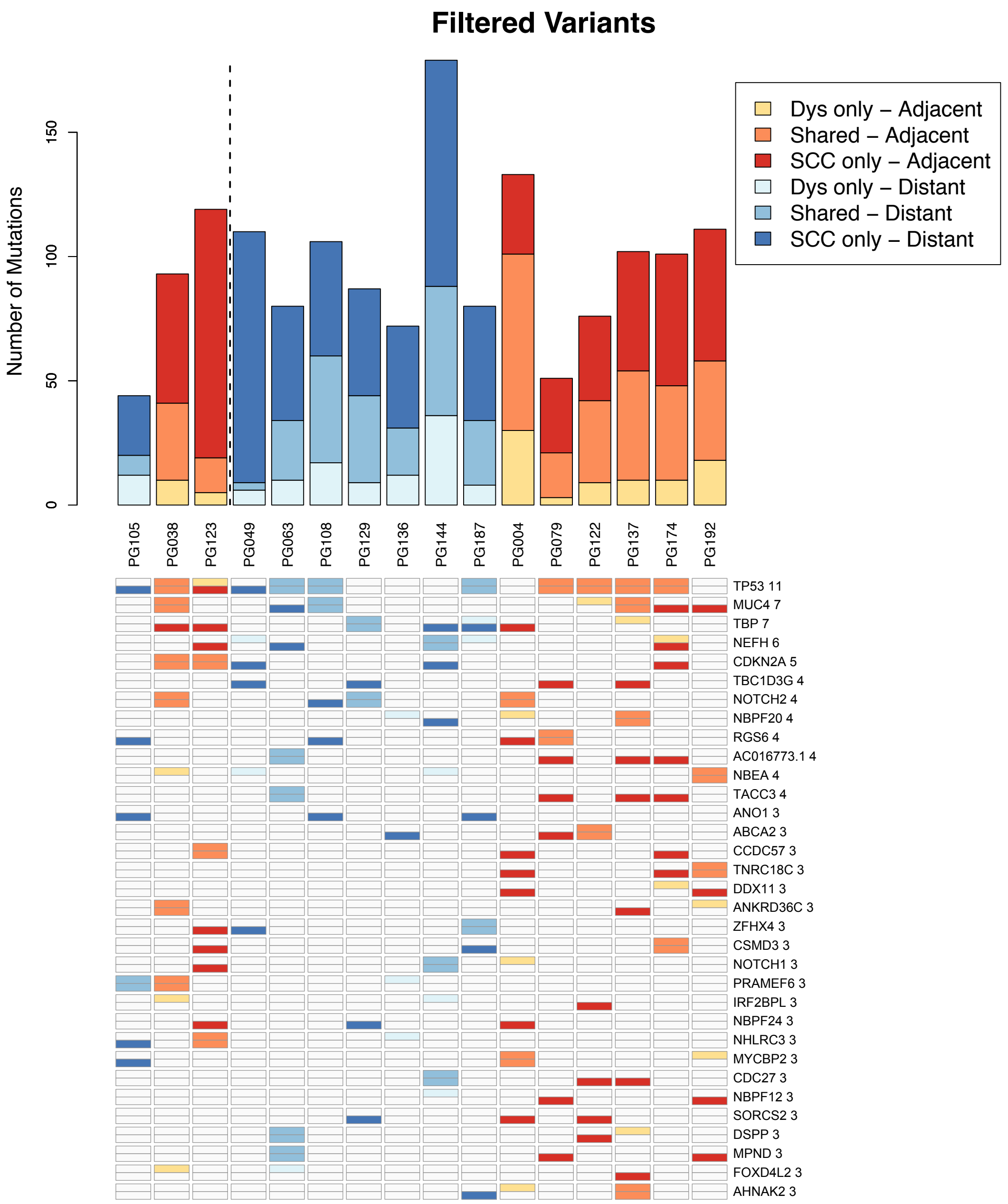

Figure S5: Comparison of shared mutation calls between and within samples. For every sample, the number of shared mutation calls with every other sample from the same and different patients was counted and the VAF noted. The calls are displayed in full, and over three different VAF ranges, showing that even at low VAFs, there were more calls shared within than between patients.

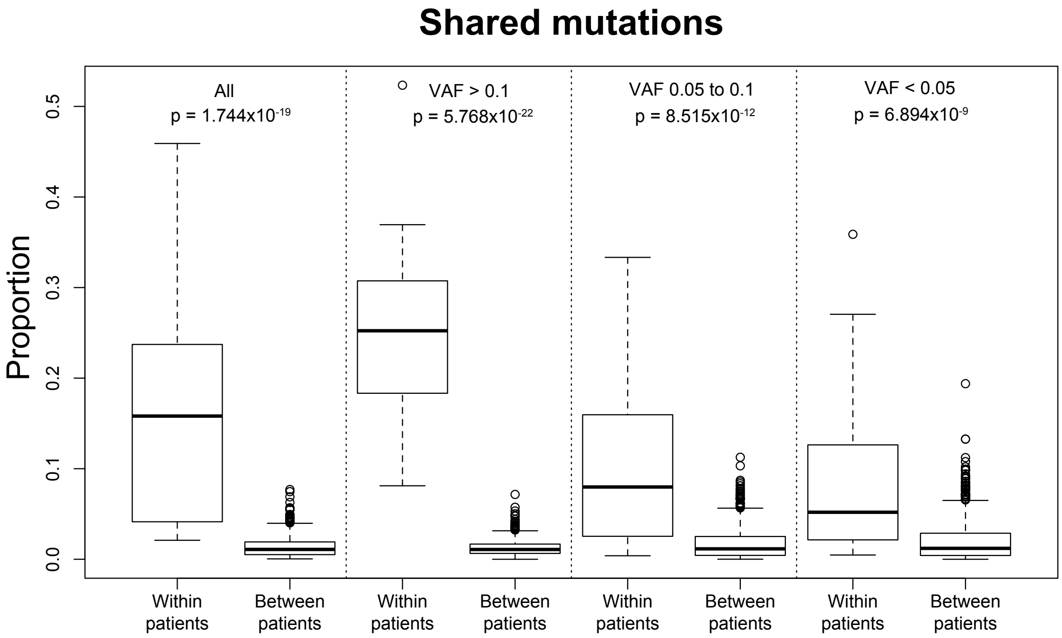

Figure S6 (following pages): Comparison of shared and private variant allele frequencies for all exome sequencing samples. For each patient, in the left plot, all putative mutations are displayed as SCC VAF versus dysplasia VAF. Private mutations are on the axes at x=0 or y=0. To make the distributions of VAFs more visible, they are displayed as histograms in the right plots. On the left, the distribution of dysplasia VAFs are shown, with the shared mutations in red. On the right the SCC VAFs are shown. For many patients, there were high numbers of putative calls with very low VAFs which may be spurious, and which make the rest of the distribution difficult to see. Therefore, the bottom two plots replicate the top plots, but only with VAFs > 0.12. For each histogram, the p-value showing the likelihood of the shared and total distributions being the same is shown.

S6a: PG004

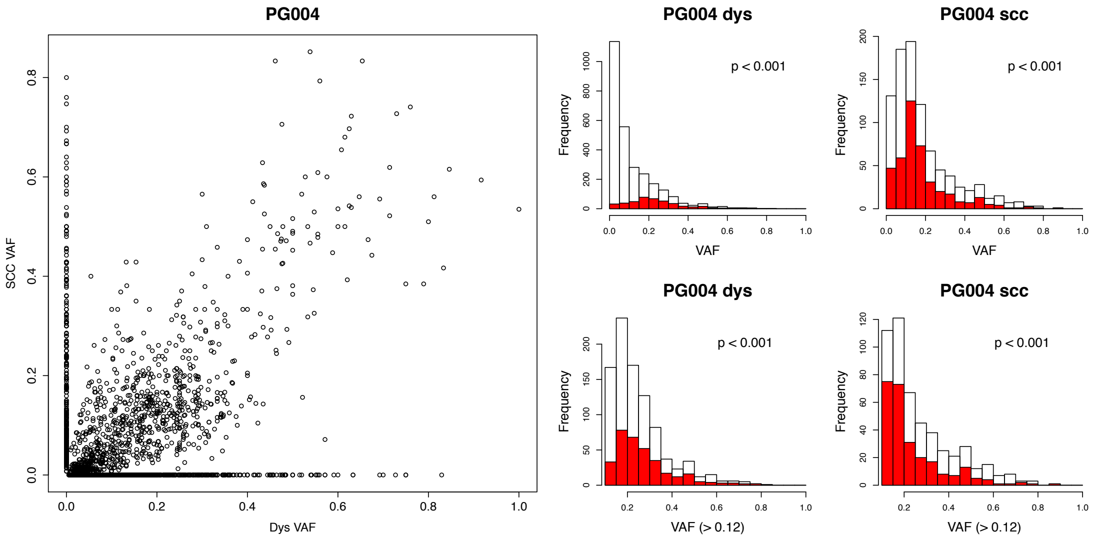

S6b: PG038

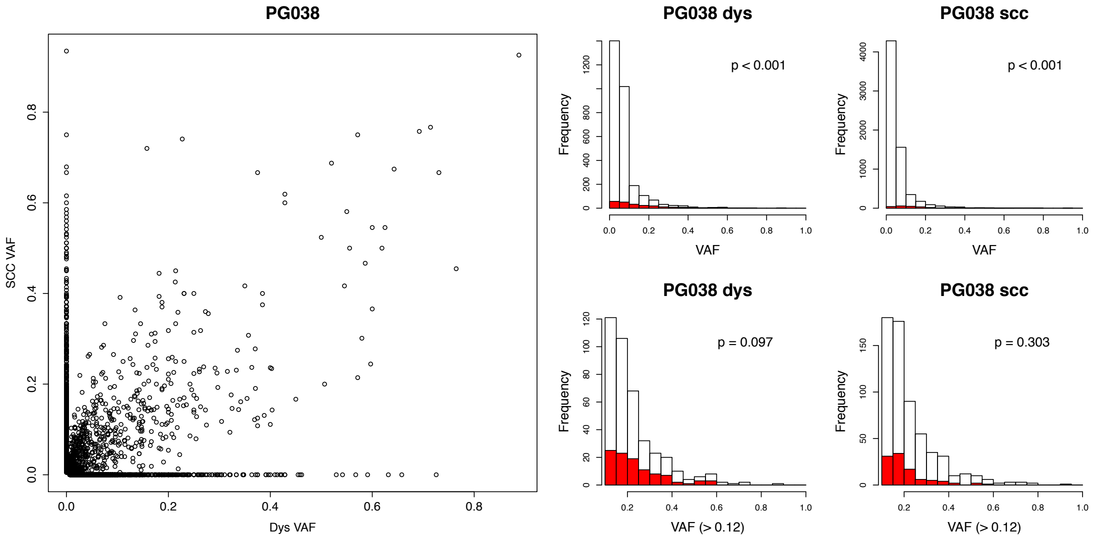

S6c: PG049

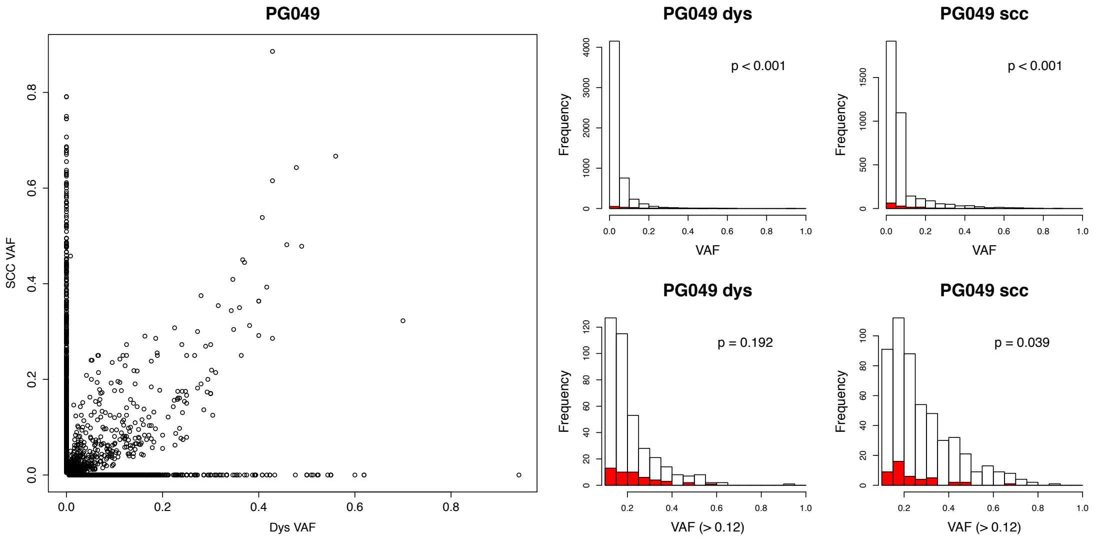

S6d: PG063

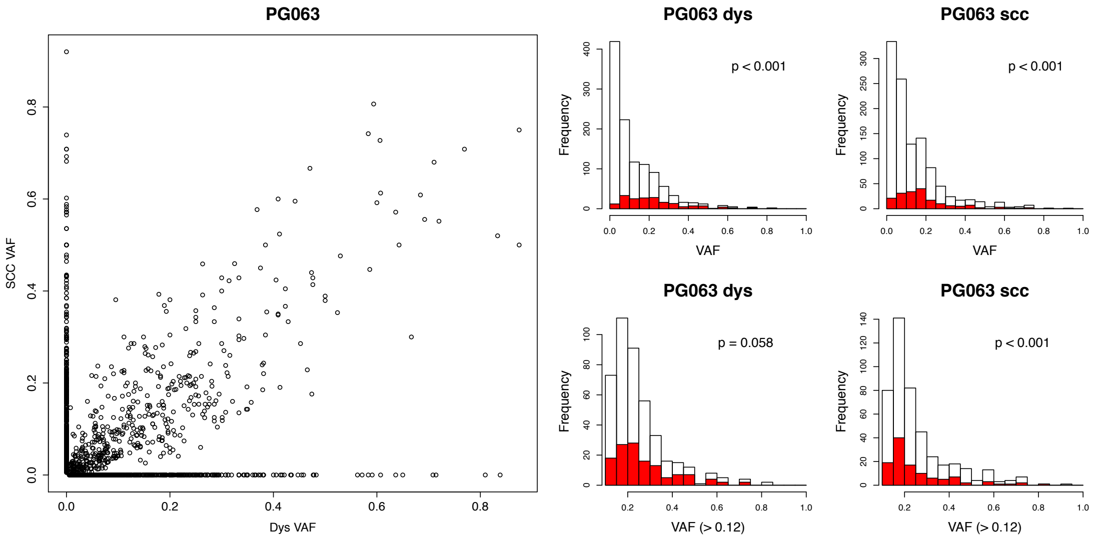

S6e: PG079

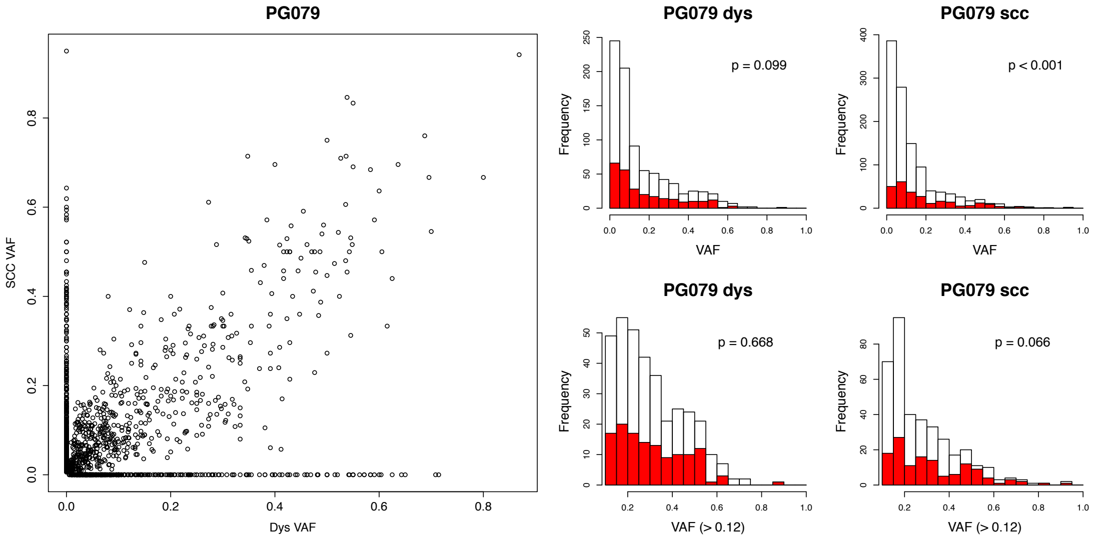

S6f: PG105

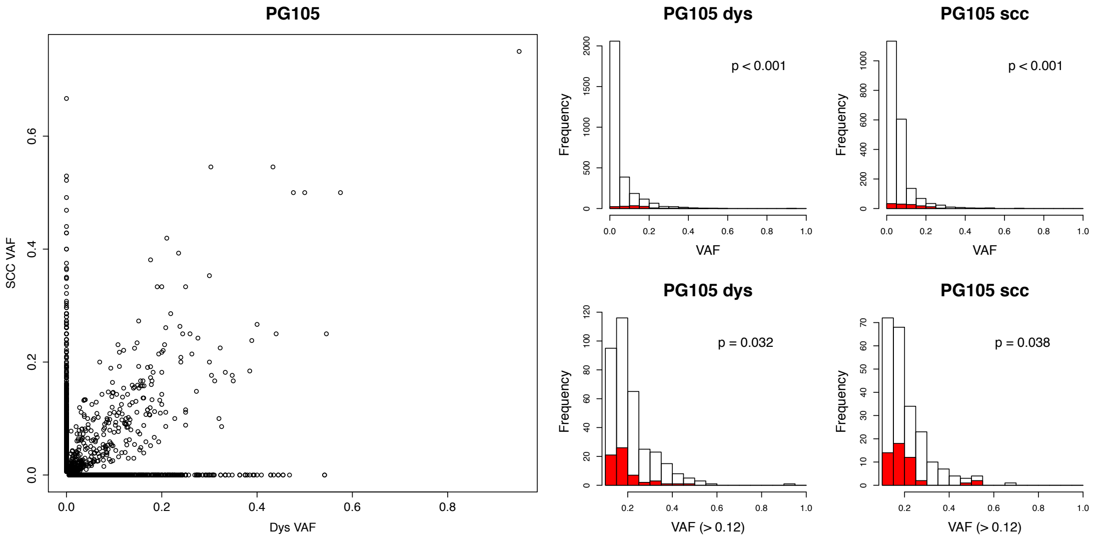

S6g: PG108

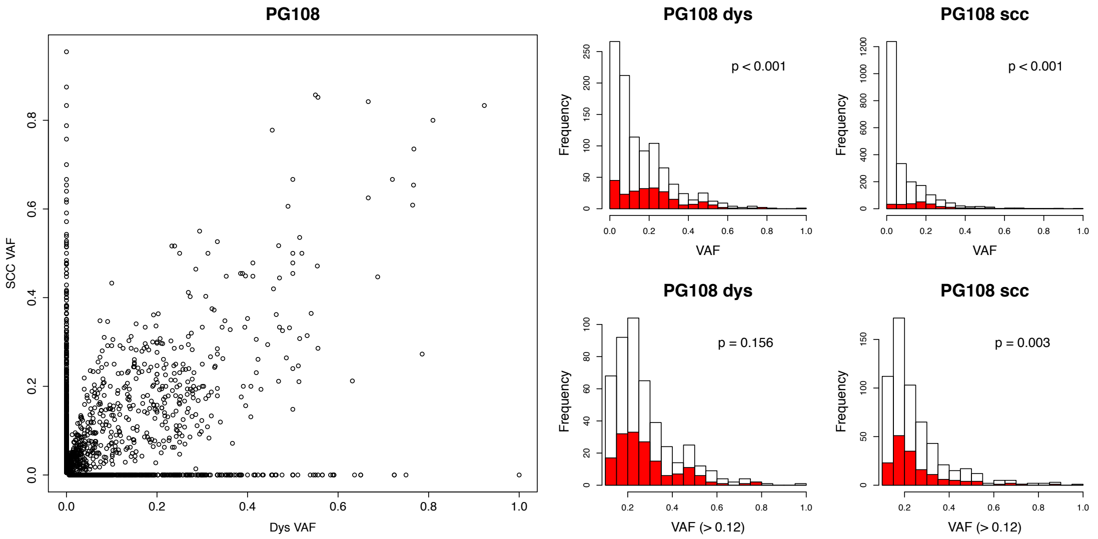

S6h: PG122

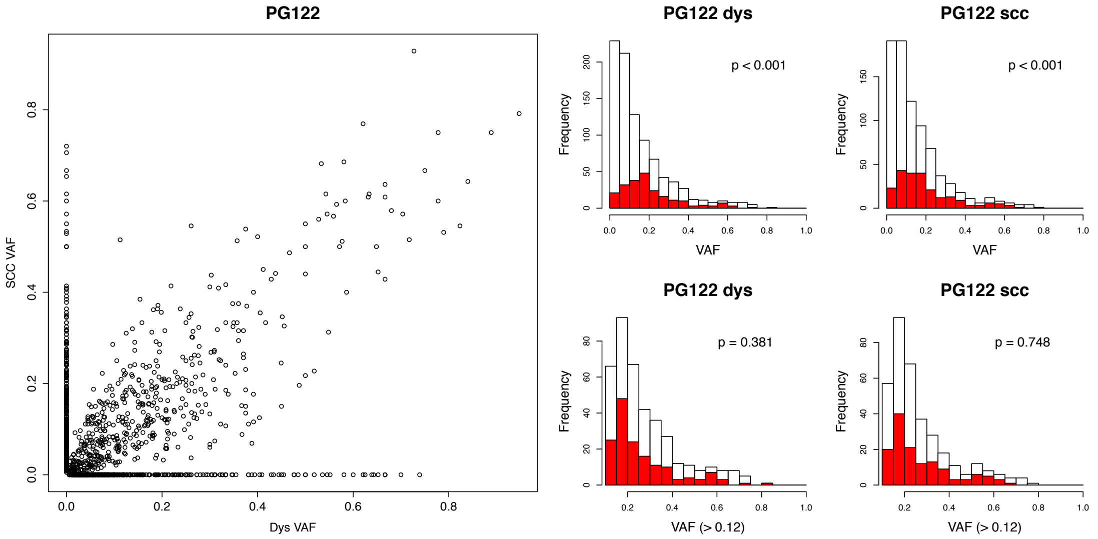

S6i: PG123

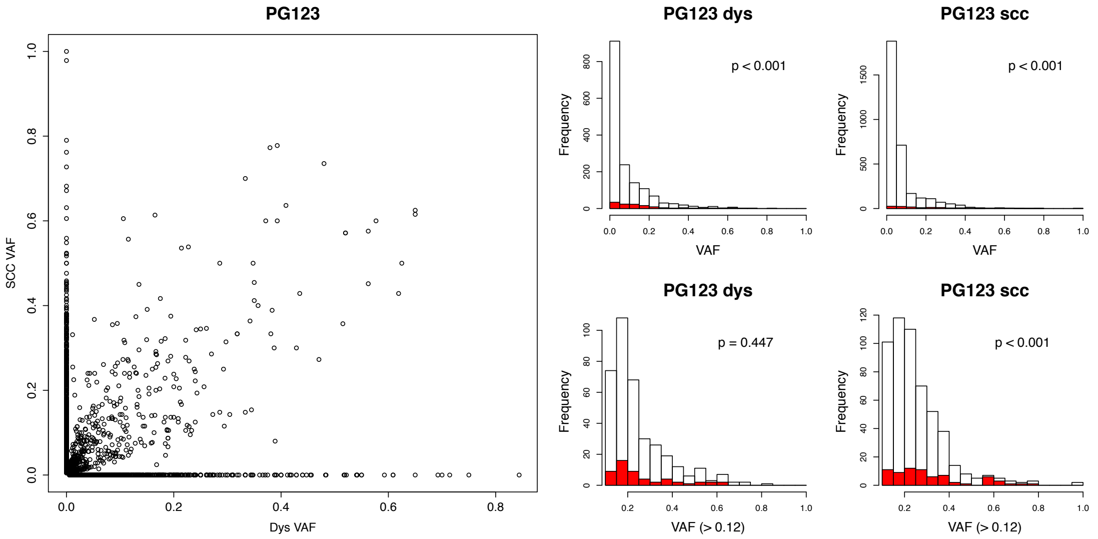

S6j: PG129

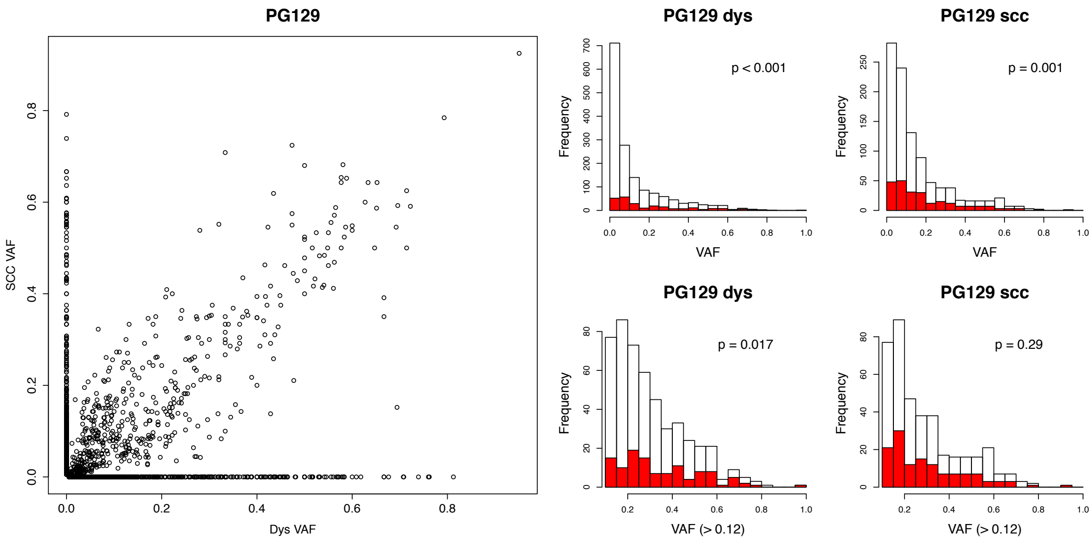

S6k: PG136

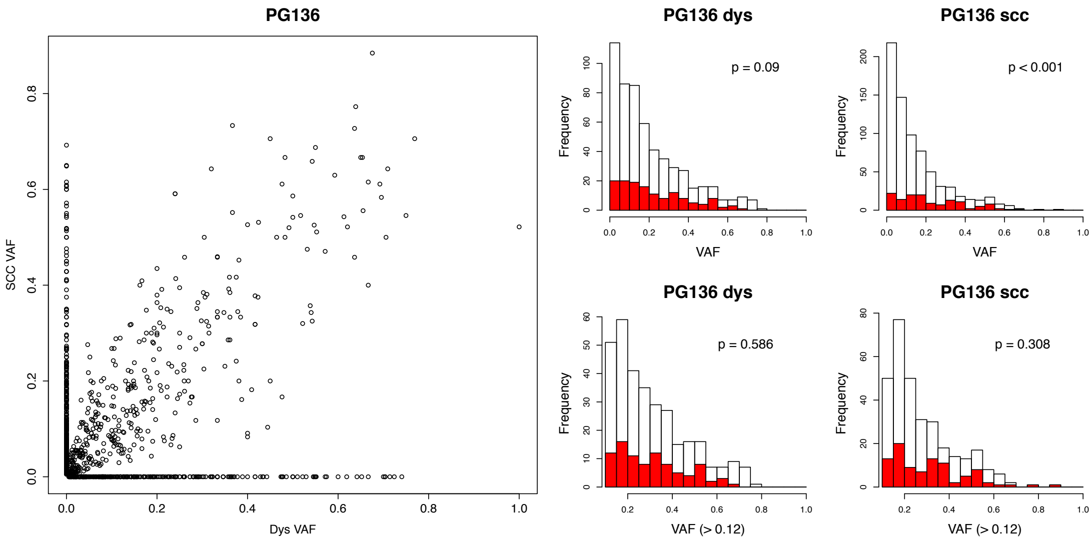

S6l: PG137

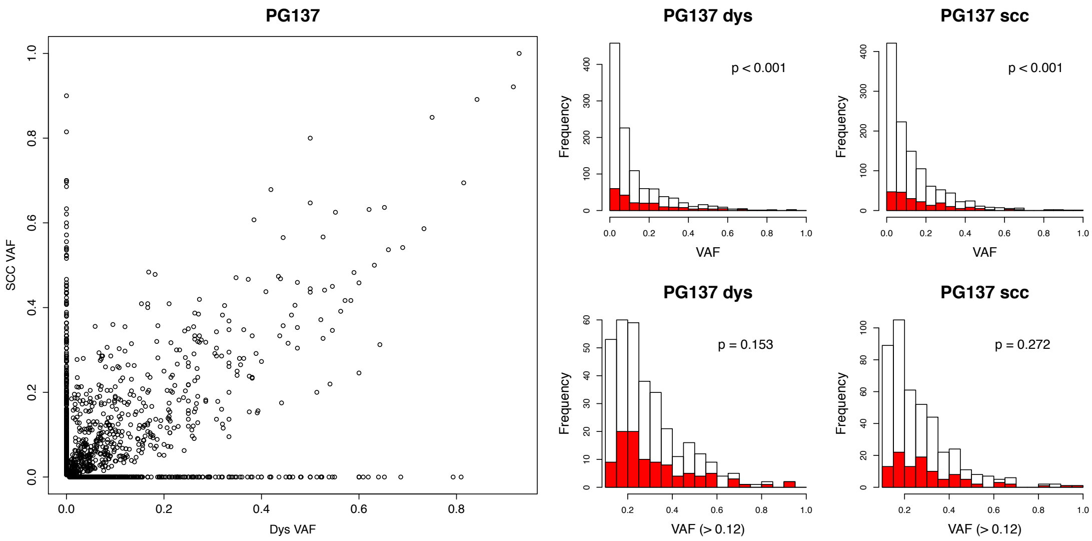

S6m: PG144

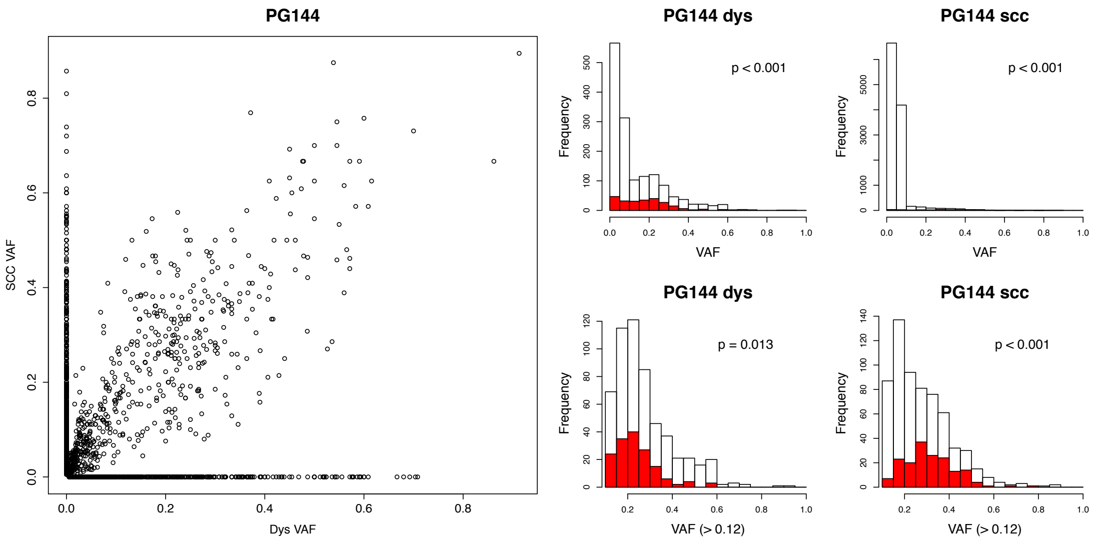

S6n: PG174

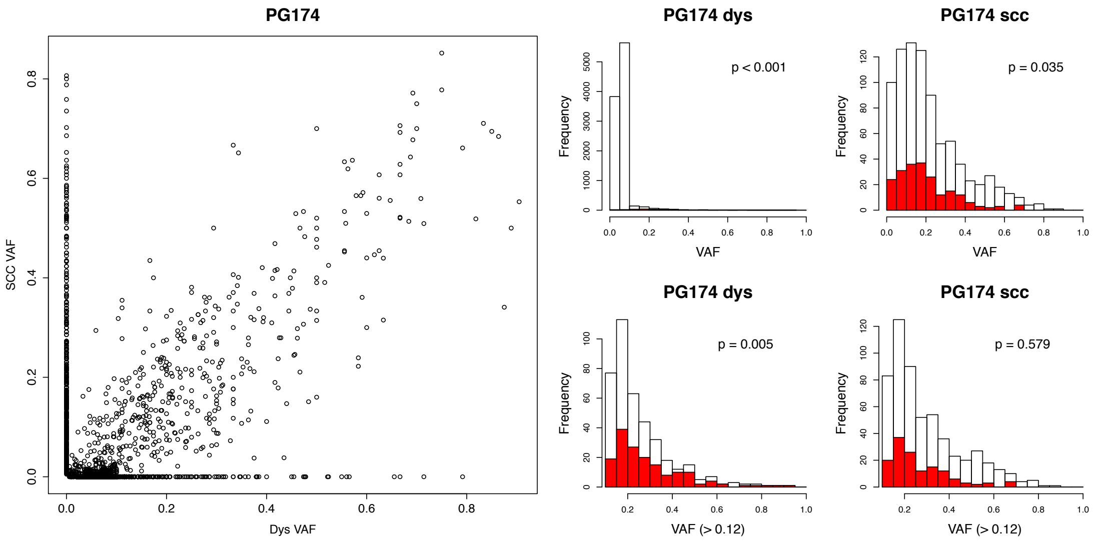

S6o: PG187

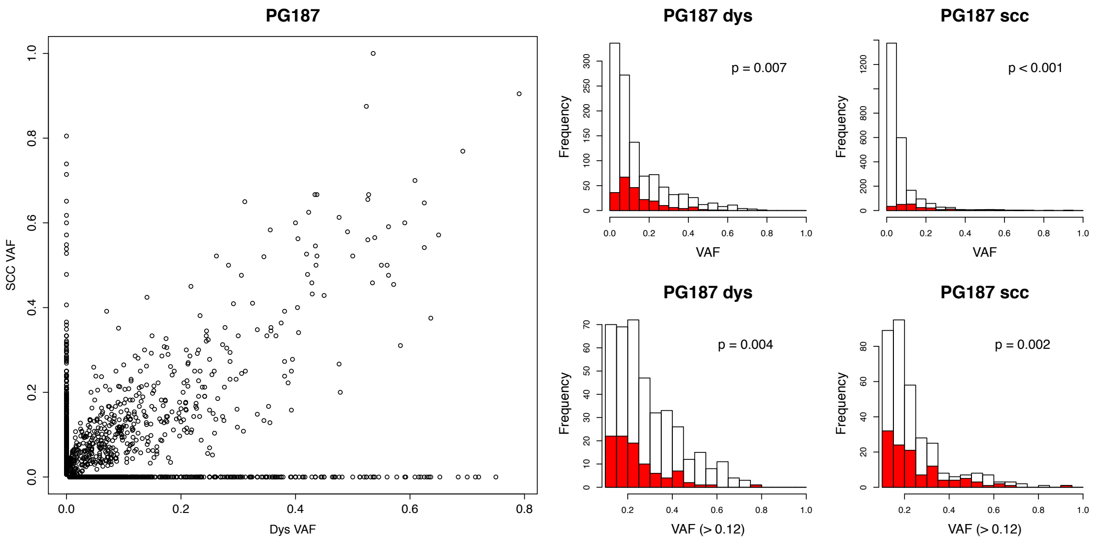

S6p: PG192

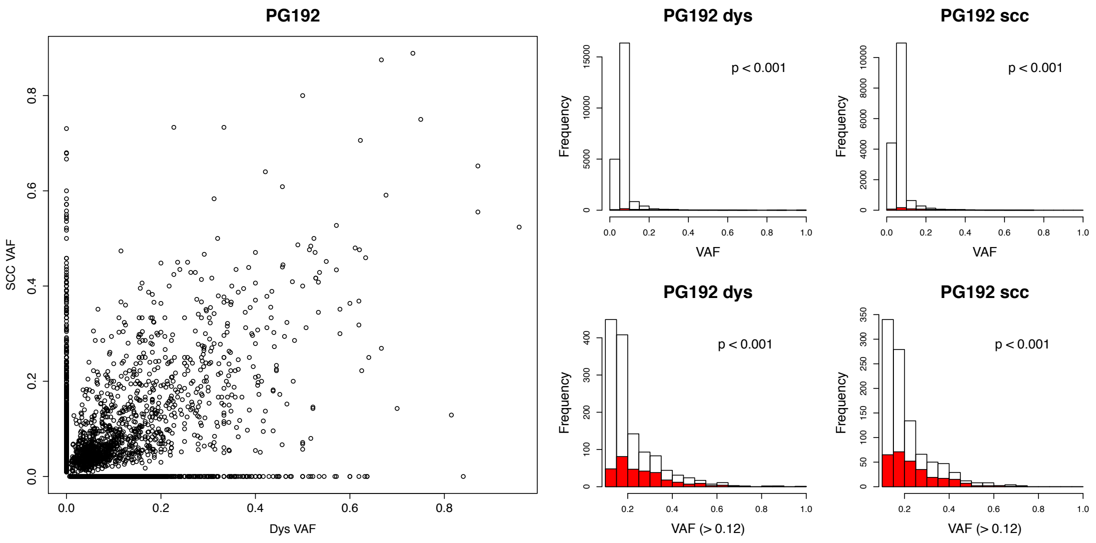

Figure S7 (following pages): Plots to test for neutral tumour evolution. For each exome sample, for all mutations with a VAF > 0.12 and < 0.24 (thereby excluding truncal mutations and spurious calls), a plot of number of cumulative mutations at a VAF versus 1/VAF is shown. An R^2 goodness of fit value is shown, with values over 0.98 being indicative of neutrality.

S7a: PG004

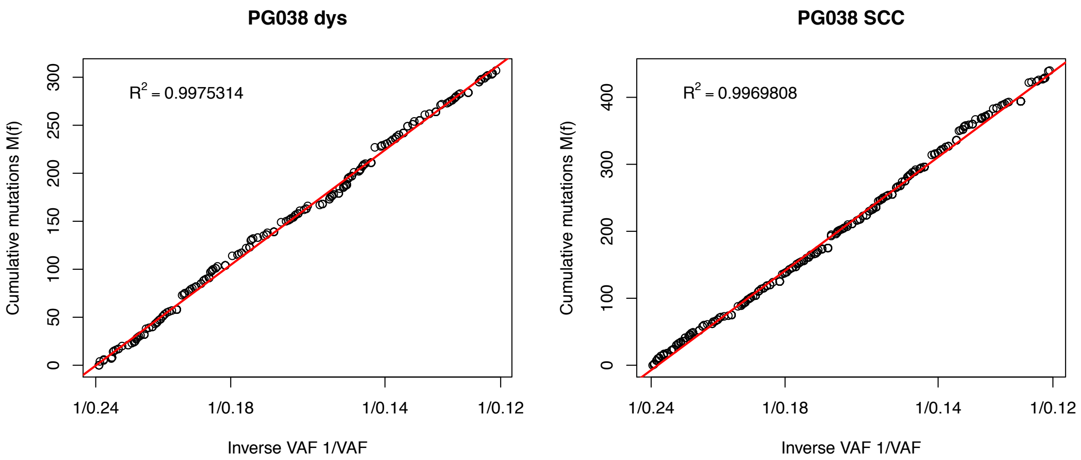

S7b: PG038

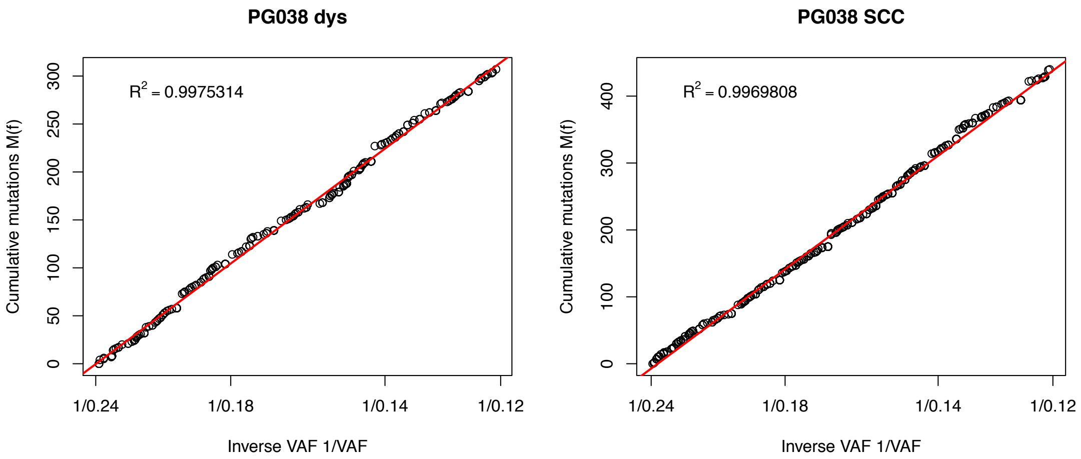

S7c: PG049

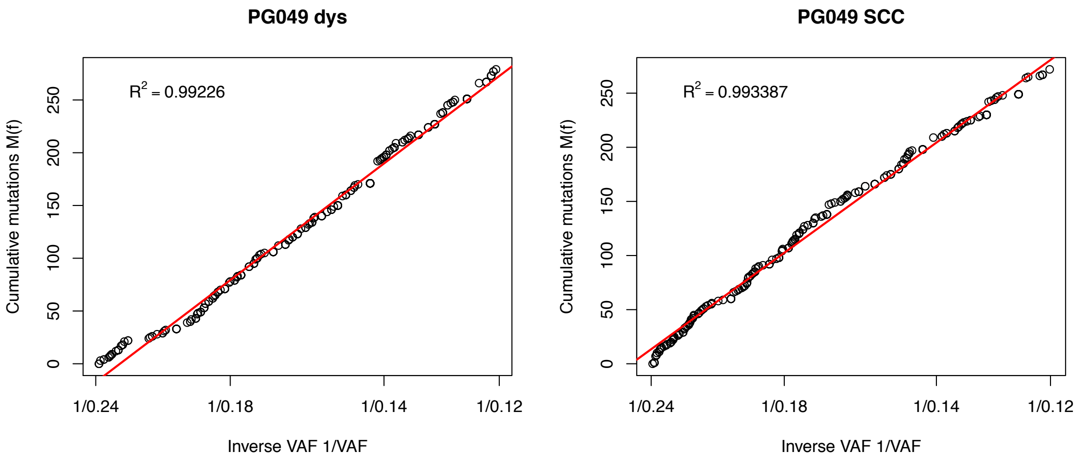

S7d: PG063

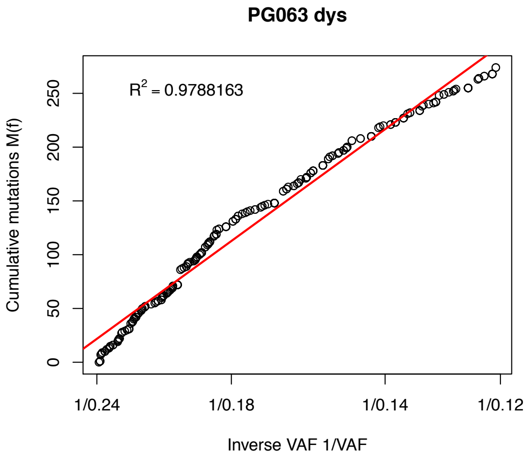

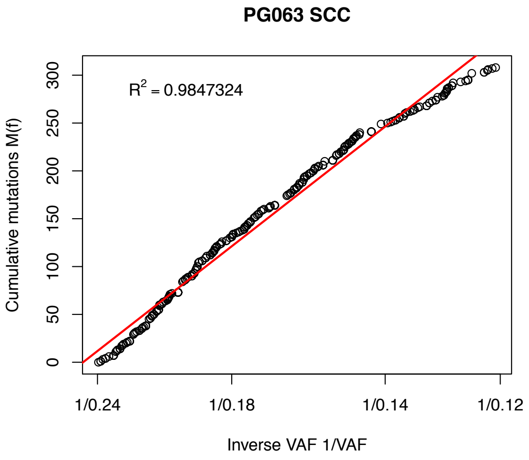

S7e: PG079

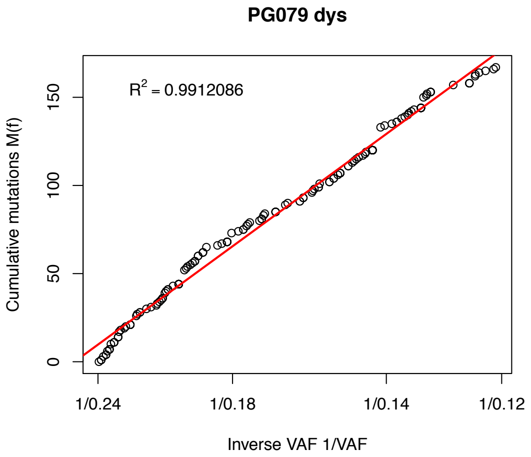

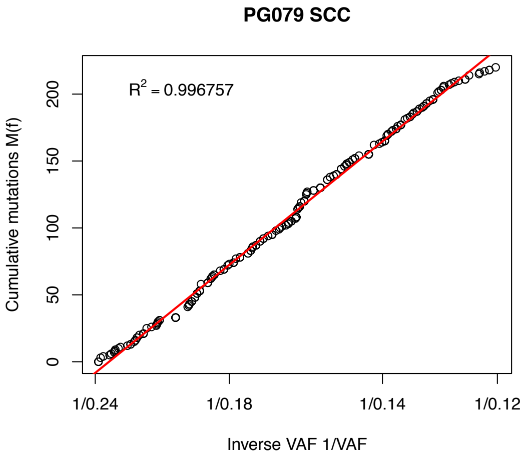

S7f: PG105

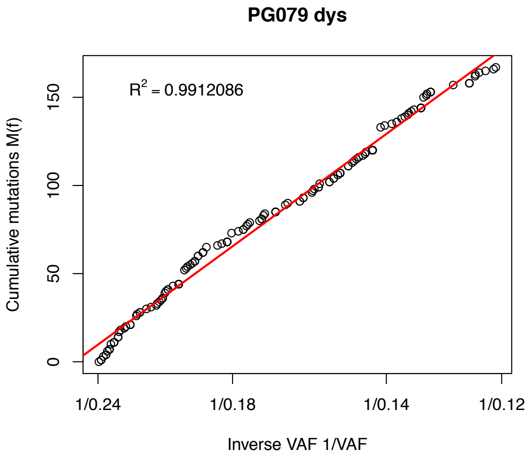

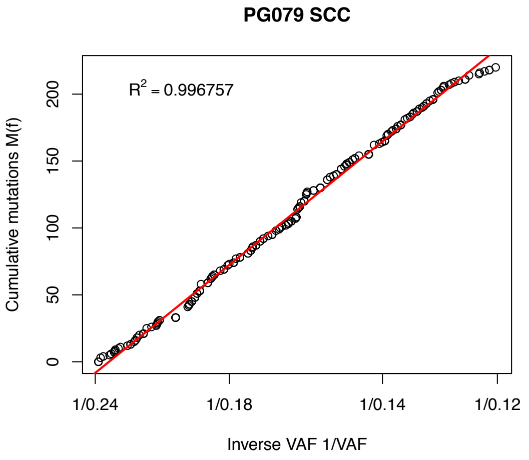

S7g: PG108

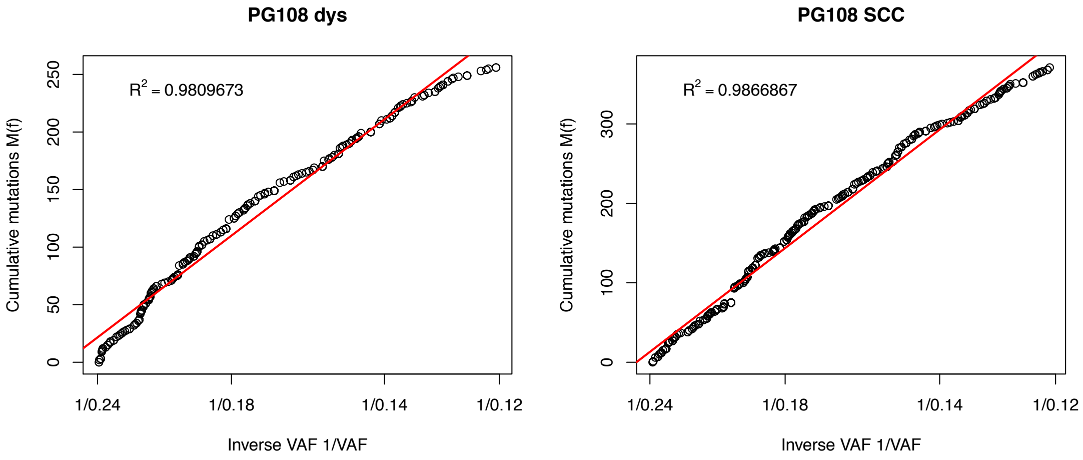

S7h: PG122

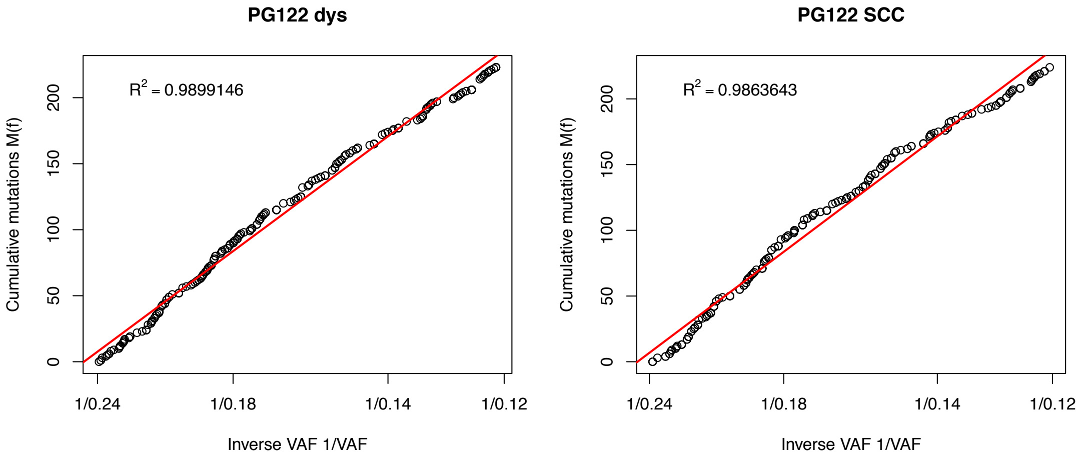

S7i: PG123

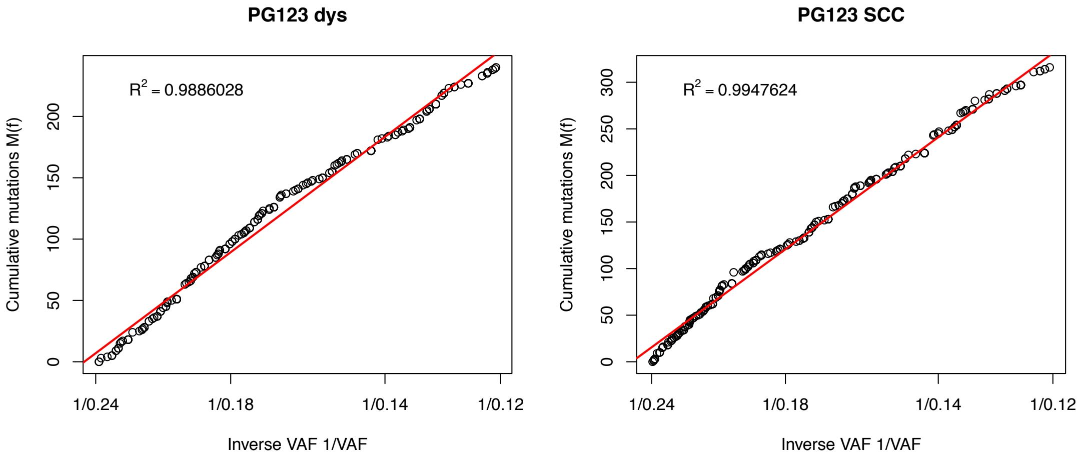

S7j: PG129

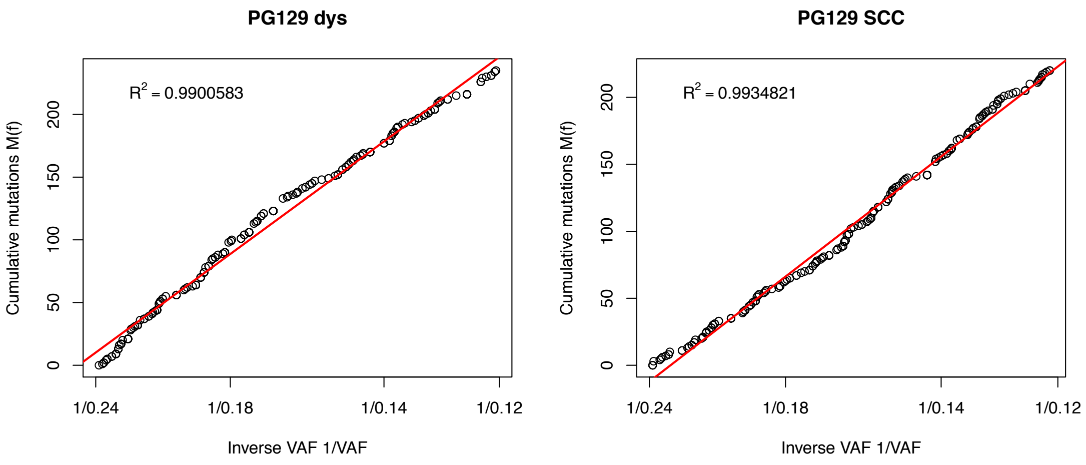

S7k: PG136

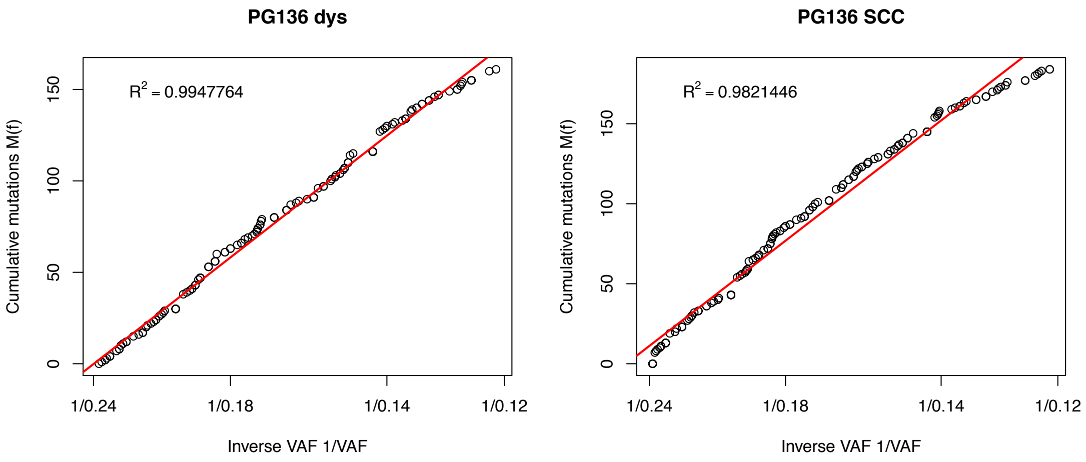

S7l: PG137

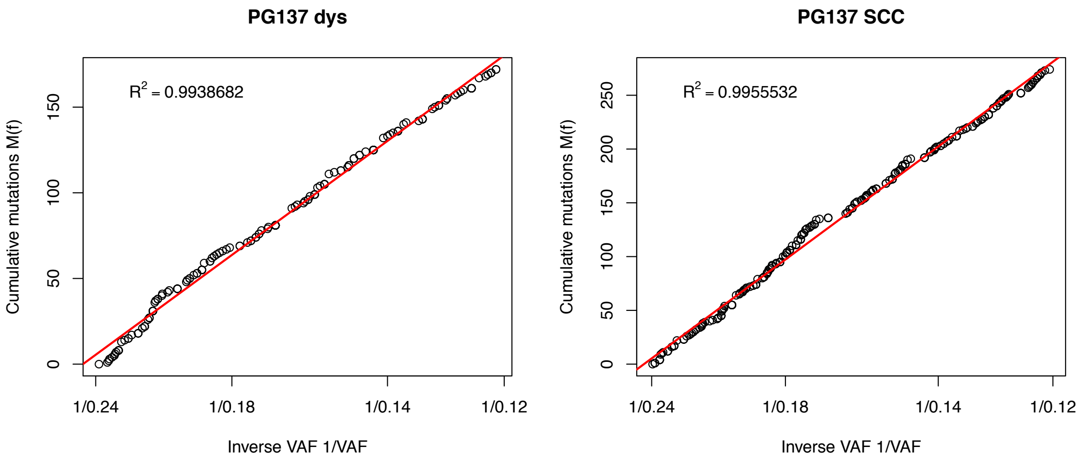

S7m: PG144

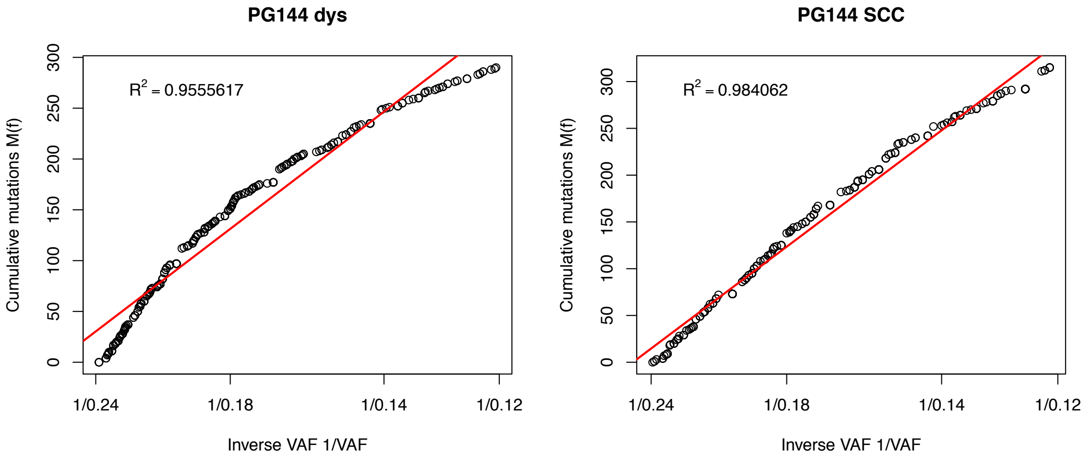

S7n: PG174

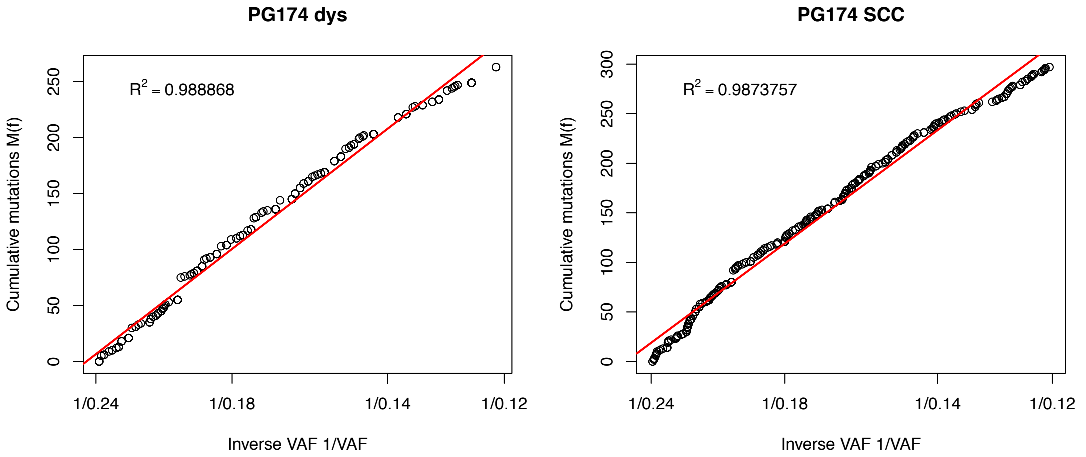

S7o: PG187

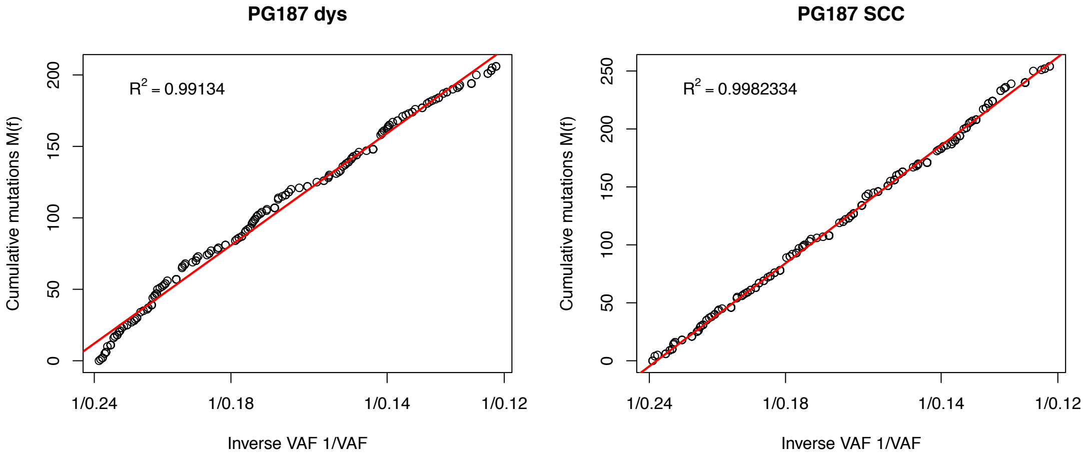

S7p: PG192

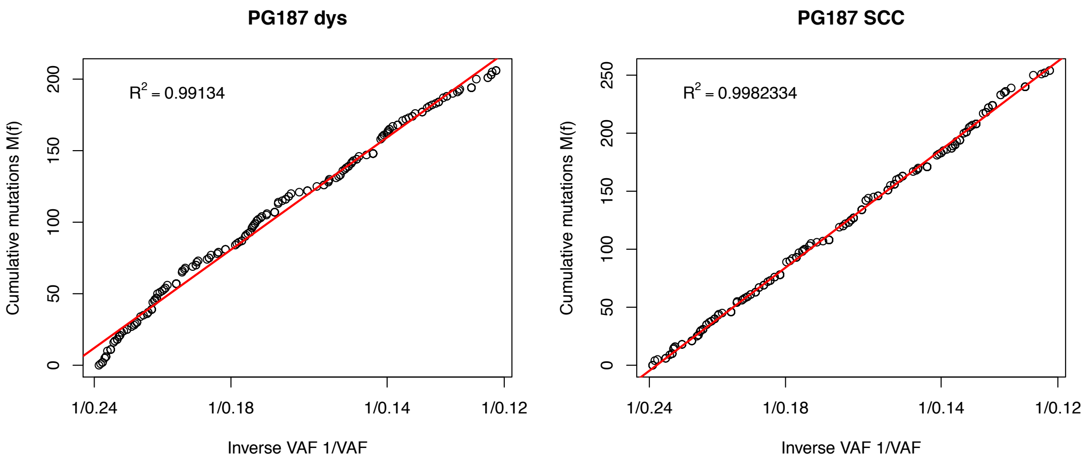
PG187 dys
R^2 = 0.99134
Cumulative mutations M(f)
0
50
100
150
200
1/0.24
1/0.18
1/0.14
1/0.12
Inverse VAF 1/VAF
PG187 SCC
R^2 = 0.9982334
Cumulative mutations M(f)
0
50
100
150
200
250
1/0.24
1/0.18
1/0.14
1/0.12
Inverse VAF 1/VAF

Figure S8: All filtered mutations in suspected cancer genes observed. The number to the right of each gene indicates the number of patients with a mutation in that gene. The colour of the bar indicates whether the mutation was in dysplasia, SCC or shared, and whether the SCC was adjacent to the SCC in that patient. The three leftmost patients had LGD, the remainder HGD.

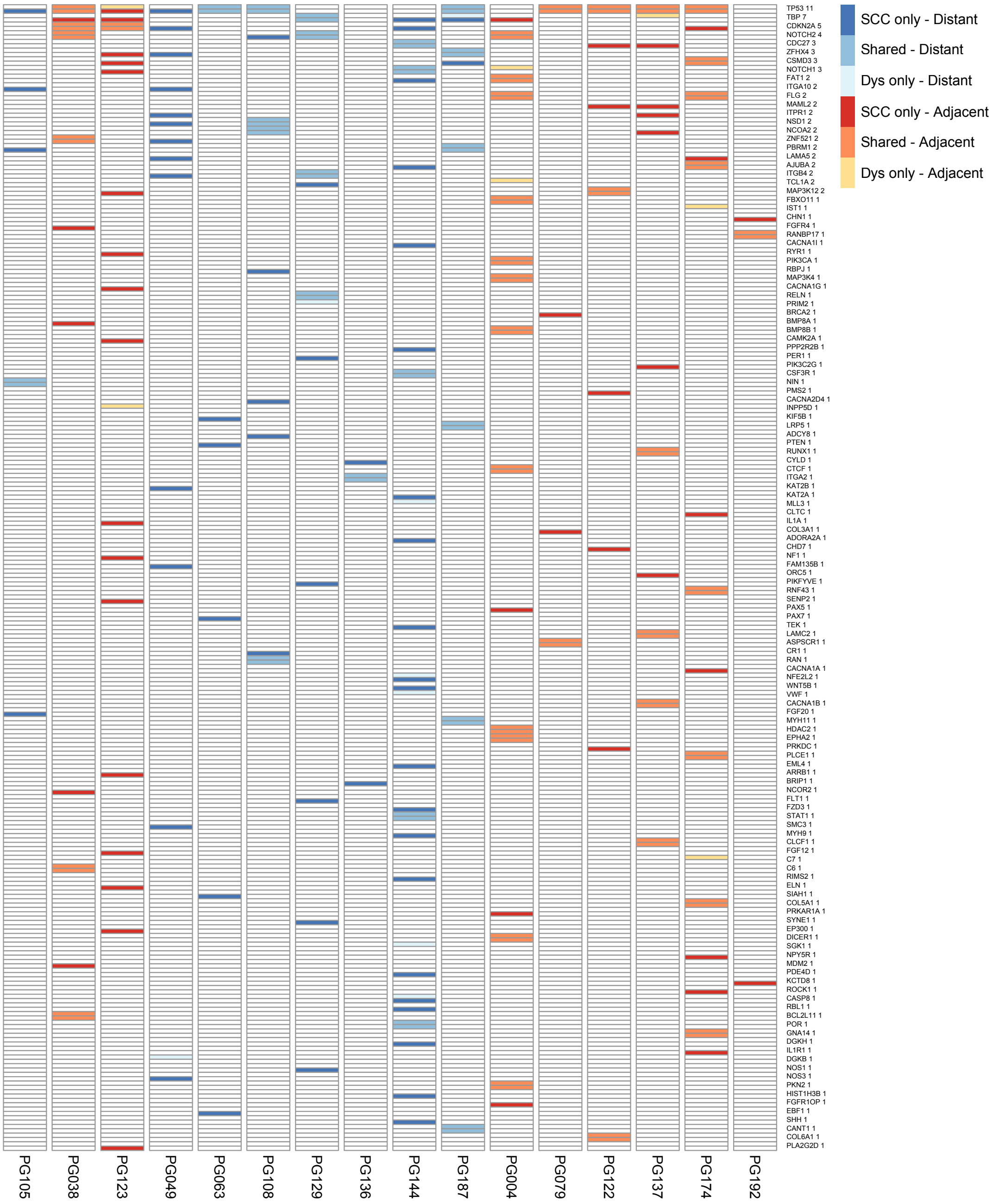

Table S3: Lists of genes mutated in 16 patients from whom exome data was obtained. Mutations were filtered by cellular frequency and effect on gene function. Mutations are classed by whether they were unique to either dysplasia or SCC sample or shared, and by whether they are in suspected cancer genes or not. If a gene is listed in more than one column for a patient, that indicates two distinct mutations in different samples.

**suspected cancer genes**		
**PG004 dysplasia only**	**PG004 shared**	**PG004 SCC only**
TCL1A	*FBXO11*	*TBP*
NOTCH1	*HDAC2*	*PRKAR1A*
EPHA2	*DICER1*	*PAX5*
	MAP3K4	*FGFR1OP*
	EPHA2	
	FAT1	
	CTCF	
	PKN2	
	PIK3CA	
	CTCF	
	FLG	
	BMP8B	
	NOTCH2	

**other genes/genomic features**		
**PG004 dysplasia only**	**PG004 shared**	**PG004 SCC only**
ACACA	*LGALS16*	*ATG2B*
RP13-60M5.2.1	*ADRA2C*	*CDYL2*
HNRNPCL1	*AC079354.1.1*	*ITSN2*
SVEP1	*LPHN2*	*AC128677.4.1*
NOL4	*TGM5*	*FCER2*
NUDT12	*PTGFRN*	*SORCS2*
MDN1	*SLC28A1*	*PRAMEF9*
RP4-635E18.6.1	*DNAH10*	*DDX11*
GABRG1	*COL15A1*	*CHID1*
ATXN1	*ACIN1*	*G2E3*
CTC-451A6.1.1	*GALNT13*	*FAM20A*
SLC40A1	*ANK3*	*LRRC48*
CST3	*CTDSP1*	*ATPAF2*
OR4C3	*TP53BP2*	*NEDD9*
OR4C3	*SOX9*	*CCDC57*
TIMM44	*OR8F1P*	*NF1P6*
CASD1	*UGT2A3*	*XXyac-YX155B6.3.1*
FCGBP	*TGM7*	*NBPF24*
RP11-231C14.4.1	*WFIKKN2*	*SORCS3*
TRAK2	*DNAH6*	*TPRX1*

NBPF20	*FAM35B*	*AMBRA1*
C15orf40	*KCNJ4*	*ECD*
DYNC1LI2	*RRP12*	*SNX29P2*
WNK1	*TMEM150C*	*KRT16P2*
AHNAK2	*RP11-59N23.1.1*	*TNRC18C*
MYO10	*STX2*	*KRT83*
NPIPL2	*RPL14*	*RGS6*
	AC107081.5.1	*SEPSECS*
	CCT4	
	EHF	
	DNAH9	
	ACTR3C	
	CECR2	
	DNMT3L	
	VPS13B	
	OVOL3	
	AFM	
	REXO1L2P	
	ATP8B1	
	ATP6V1B2	
	DLG5	
	SEMA3A	
	LRRC16B	
	UNKL	
	RADIL	
	CSMD1	
	ITGAL	
	C22orf25	
	METRN	
	MYCBP2	
	CYP1B1	
	C9orf43	
	SIDT1	
	GGN	
	ZCWPW1	
	SNX2	
	FER	
	TNFRSF25	

**suspected cancer genes**		
**PG038 dysplasia only**	**PG038 shared**	**PG038 SCC only**
	BCL2L11	*FGFR4*
	CDKN2A	*NCOR2*
	C6	*TBP*
	NOTCH2	*MDM2*

	NOTCH2	*BMP8A*
	ZNF521	
	TP53	

**other genes/genomic features**		
**PG038 dysplasia only**	**PG038 shared**	**PG038 SCC only**
TPSAB1	*MCTP1*	*NARG2*
IRF2BPL	*AC008103.5.1*	*SNX24*
RP3-377D14.1.1	*SMYD4*	*DPYD*
FOXD4L2	*MEF2A*	*PABPC1*
RP11-534G20.3.1	*MERTK*	*GCKR*
RP11-146D12.2.1	*EXOC1*	*RP11-368J21.1.1*
MUC4	*BIRC6*	*EIF4G3*
FAM90A1	*NCAN*	*DNAH12*
FAM22G	*MUC4*	*MFHAS1*
NBEA	*APOB*	*ZDHHC14*
	ZAN	*F11R*
	LAPTM4A	*PCDHGB2*
	TMEM88	*SNAPC2*
	MUC4	*IRF5*
	GOLGA8G	*FAM193A*
	ANKRD36C	*RIC8B*
	ZSCAN5B	*PTPN4*
	NRP2	*SEC14L5*
	FUZ	*SLC25A29*
	PRAMEF6	*CNOT10*
	MAGEL2	*RALGAPB*
	PCDHB18	*PCSK1*
	SNAP25	*DNAH7*
	RORB	*DNMT1*
		MEPCE
		EGR2
		TSC22D2
		C14orf23
		BMPR1B
		SLC35B1
		WDPCP
		MBTPS1
		TDRD12
		NBPF10
		TTC39C
		ANKLE2
		PEBP4
		RP11-459E5.1.1
		RP11-69E11.4.1

		HTN1
		GMEB1
		AC100803.1.1
		MST1
		RP11-88I21.2.1
		UPF3A
		C8B
		SPATA8

**suspected cancer genes**		
**PG049 dysplasia only**	**PG049 shared**	**PG049 SCC only**
DGKB		*ITGA10*
		ITGB4
		ITPR1
		NSD1
		CDKN2A
		NOS3
		SMC3
		KAT2B
		ZFHX4
		TP53
		LAMA5
		ZNF521
		FAM135B

**other genes/genomic features**		
**PG049 dysplasia only**	**PG049 shared**	**PG049 SCC only**
MED31	*ATP8A1*	*CCDC33*
NBEA	*MDC1*	*CTNS*
NEFH	*LMAN1*	*KCNQ3*
ANKLE1		*C6orf132*
AC004967.7.1		*DYNC1I1*
		PTPRQ
		SSH1
		CNTLN
		RHOT2
		GALK1
		IL27RA
		COL13A1
		DTNB
		GALNT13
		CATSPER1
		WIPF1
		EYA4
		UBR3

			TRGC2
			CFHR4
			AP1G2
			RP11-66N24.3.1
			USP6NL
			HIST1H4F
			GRID2
			SOD2
			WTAP
			USF1
			FAM75A7
			ATP13A5
			TNRC6B
			MPP2
			TRDN
			NARS
			GRHPR
			PHYH
			IGHV4-34
			SPG11
			FGD6
			SOD2
			PENK
			NAT8
			RWDD2B
			STXBP4
			C2orf71
			TRAP1
			OR2A4
			DYNC1I1
			ARMC9
			SARDH
			HERC2
			PRPF4B
			RFPL4A
			C6orf103
			GFRA1
			BTBD8
			BRD7
			CCDC80
			PRUNE2
			SLC12A9
			TMEM178
			SLC24A6
			IL27RA
			DDX60L

		GMEB2
		TXNDC16
		KPNA2
		CENPM
		SNAP91
		TRPC4
		SUSD1
		PSG4
		CAMKMT
		PCSK5
		TBC1D3G
		CDH24
		RNF213
		AKAP6
		RP11-1220K2.2.1
		MTFR1
		TCF25
		FSTL5
		GULP1
		ASNS
		PRKD3
		DMRTA2
		SACS
		FOLR4

**suspected cancer genes**		
**PG063 dysplasia only**	**PG063 shared**	**PG063 SCC only**
	TP53	*KIF5B*
		PTEN
		SIAH1
		PAX7
		EBF1

**other genes/genomic features**		
**PG063 dysplasia only**	**PG063 shared**	**PG063 SCC only**
FOXD4L2	*MPND*	*POTEF*
COL12A1	*SLC10A2*	*CERS3*
OR7G1	*LZTR1*	*PZP*
ZNF812	*OR9I1*	*MUC4*
PNPLA8	*CWC15*	*HELZ*
CNBD1	*PNPLA7*	*BTG4*
HNRNPC	*DSPP*	*NECAB3*
PRR14	*RP11-742B18.1.1*	*DPF2*
CASD1	*TCP10L2*	*PSD*
PUM1	*ALMS1*	*RP1-170O19.2.1*

	MIER2	*CLMP*
	PARP4P2	*PLEKHH3*
	RP11-382J12.1.1	*SDHAP1*
	SRSF4	*FAM71E2*
	RBM39	*AC004019.10.1*
	AC016773.1	*CECR2*
	TACC3	*NEFH*
	UNC45A	*AL031590.1*
	MYH8	*RRN3P1*
	RPL6P12	*SLC10A6*
	SH3RF1	*ACACA*
	ZNF644	*MESP2*
	FAM86C1	*KRT37*
		FNDC3A
		IQSEC3
		TGIF2
		ZNF737
		AMPH
		C1D
		GOLGA8H
		CD58
		SAFB2
		STARD9
		USP33
		RP11-1220K2.2.1
		SNHG5
		PRKAG1
		RP11-386G11.5.1
		TRPV4
		EPHX4
		LITAF

**suspected cancer genes**		
**PG079 dysplasia only**	**PG079 shared**	**PG079 SCC only**
	ASPSCR1	*COL3A1*
	TP53	*BRCA2*

**other genes/genomic features**		
**PG079 dysplasia only**	**PG079 shared**	**PG079 SCC only**
PRAMEF15	*RGS6*	*MPND*
RP11-144A16.1.1	*ARL4A*	*ARHGEF40*
SLC25A28	*MUC12*	*PCGF6*
	GUCY2C	*PPP1R13B*
	RP11-174G6.1.1	*PABPC1*
	PDE9A	*KIRREL2*

	AC006465.3.1	*NPHS1*
	PCDH8P1	*KRTAP5-5*
	PPP1R16B	*GLG1*
	FAM22F	*UIMC1*
	RP11-356C4.2.1	*PABPC1*
	IRX6	*HUNK*
	NCOA3	*RP11-1348G14.2.1*
	ZNF799	*NBPF12*
	PRAMEF13	*TBC1D3G*
	UPF3A	*DOCK8*
		EFCAB5
		GALNT7
		CAPN12
		POLR3H
		ABCA2
		HNRNPC
		TACC3
		AC016773.1
		PPFIA2
		OFCC1
		RCN3
		NBPF12

**suspected cancer genes**		
**PG105 dysplasia only**	**PG105 shared**	**PG105 SCC only**
	NIN	*ITGA10*
		PBRM1
		FGF20
		TP53

**other genes/genomic features**		
**PG105 dysplasia only**	**PG105 shared**	**PG105 SCC only**
MTMR11	*EPS8*	*EPHA10*
KARS	*AC093818.1.1*	*MAST1*
AC026150.9.1	*TUBA3E*	*NHLRC3*
POM121C	*RNF17*	*RBMXL1*
CCDC69	*PRAMEF6*	*IFT43*
ZSWIM6	*DDA1*	*RGS6*
PANK3	*RP11-286O18.1.1*	*HDC*
PROSER1		*TPRXL*
AMY2A		*CCT5*
GOLGA6L2		*KIAA1107*
AC026150.9.1		*GFPT2*
FAM90A1		*BX004987.5.1*
		TRIM49L2

		ANO1
		MYCBP2
		C19orf44
		CALR3
		XXbac-BPG55C20.1.1
		SLC45A4
		GDAP1L1

**suspected cancer genes**		
**PG108 dysplasia only**	**PG108 shared**	**PG108 SCC only**
	NSD1	*RBPJ*
	RAN	*CR1*
	NCOA2	*CACNA2D4*
	TP53	*ADCY8*
		NOTCH2

**other genes/genomic features**		
**PG108 dysplasia only**	**PG108 shared**	**PG108 SCC only**
OVGP1	*OR6M1*	*ROBO2*
TMEM200B	*SERINC2*	*RP11-88K11.1.1*
CDK13	*CDH8*	*RP11-257K9.6.1*
NOD1	*ZNF48*	*FAM182A*
TASP1	*SIPA1L1*	*PREX2*
TMEM200C	*FAM194B*	*AC069513.3.1*
GLIS3	*SPAG17*	*SDHAP2*
ABI3BP	*LCT*	*VPS41*
RP11-1000B6.4.1	*INTS6*	*SEC16B.1*
CTAGE8	*TRPM5*	*KIAA0664*
KRTAP4-5	*MCF2L*	*ABCB10P1*
TECPR1	*IQCK*	*BDP1*
NBPF16	*TRAPPC6A*	*TBC1D21*
RP11-1000B6.4.1	*PPP1R12A*	*C16orf62*
FAM75A7	*SPECC1L*	*ANO1*
RP11-1000B6.4.1	*KB-1896H10.1.1*	*ATG9A*
ARFIP2	*EHMT1*	*SPAG17*
	PRR19	*CDC42EP1*
	GRB14	*IL17RA*
	PIWIL4	*DGCR14*
	COPZ2	*SLC45A4*
	QTRT1	*FAM86B1*
	ACOT11	*PPAT*
	MPRIP	*ZNF653*
	MAP1A	*SYCP2*
	METTL7A	*KRTAP5-11*

	CALCOCO1	*OR7E87P*
	SLC30A5	*AP000867.1*
	SCPEP1	*CROCCP2*
	MARCH1	*C8orf80*
	KRTAP13-4	*PAX1*
	CRISP1	*POLR1C*
	ESYT2	*HAGH*
	PRSS38	*TGFB1I1*
	ZBTB41	*LCOR*
	MUC4	*SEC14L5*
	LRRC43	*CSMD1*
	ANXA13	*ACTR8*
	PLAGL1	*RGS6*
		AXDND1
		RASEF

**suspected cancer genes**		
**PG122 dysplasia only**	**PG122 shared**	**PG122 SCC only**
	COL6A1	*PMS2*
	MAP3K12	*MAML2*
	TP53	*CHD7*
		PRKDC
		CDC27

**other genes/genomic features**		
**PG122 dysplasia only**	**PG122 shared**	**PG122 SCC only**
RP11-613M5.2.1	*DENND1A*	*PGM2L1*
ZNF98	*ZNF879*	*ADCK2*
SDCCAG8	*MIDN*	*RP4-726N20.2.1*
RP11-47A17.2.1	*C21orf56*	*NDUFB2*
MUC4	*GPR98*	*NR6A1*
RP13-395E19.1.1	*SLC26A1*	*WDR41*
MUC4	*UTP11L*	*IRF2BPL*
RP5-977B1.11.1	*AGTPBP1*	*FAF1*
ACAD10	*CERS3*	*NARG2*
	EYS	*SPDYE6*
	IL16	*SUSD2*
	TMCO4	*ZFPM1*
	FAM195B	*RP11-21B21.4.1*
	TNIK	*TMEM145*
	BEST2	*SLCO4A1*
	TTBK1	*DSPP*
	GRID2IP	*RP11-742B18.1.1*
	AC100803.1.1	*DNAJB11*
	ABCA2	*KCTD1*

	CPSF1	*CCDC62*
	FAM13A	*DZANK1*
	DNAH5	*MRVI1*
	SPIRE2	*RP11-460N11.1.1*
	LPHN3	*SIRT2*
	UGT2B28	*ZFP91*
	C2orf18	*ZFP91-CNTF*
	RP11-88I21.2.1	*RP11-536C10.13.1*
	FHOD1	*SORCS2*
	PAPPA	*RP11-114H24.4.1*
	ASGR2	

**suspected cancer genes**		
**PG123 dysplasia only**	**PG123 shared**	**PG123 SCC only**
INPP5D	*CDKN2A*	*CACNA1G*
TP53		*CSMD3*
		RYR1
		ZFHX4
		ELN
		NOTCH1
		MAP3K12
		TP53
		FGF12
		PLA2G2D
		ZFHX4
		EP300
		NF1
		TBP
		ARRB1
		IL1A
		SENP2
		CAMK2A

**other genes/genomic features**		
**PG123 dysplasia only**	**PG123 shared**	**PG123 SCC only**
DNMT3L	*APBB1IP*	*DOCK9*
ANKIB1	*DUOX1*	*LRIT3*
IRF2	*NCOA5*	*CTNND1*
	GCGR	*RP11-492D6.3.1*
	ATP1B3	*ZCRB1*
	ABCA8	*NBPF24*
	NHLRC3	*CECR1*
	SGSM1	*CLK3*
	GGT7	*CNOT1*
	ZNF516	*EIF3L*

		CCDC57	*C10orf12*
		MYH13	*EIF3L*
		CNOT1	*GGA3*
			BTN1A1
			XIRP2
			AL583842.6.1
			DNAH11
			KIAA1429
			ATP10B
			NBPF24
			OR4Q2
			SLC26A3
			SLC43A2
			SF3B2
			KLHL14
			CCDC58
			CLDN23
			SHANK2
			COPS3
			IGSF22
			HAMP
			SFSWAP
			CTB-111H14.1.1
			ZNF804A
			BPIFB4
			RGS22
			MCM9
			NEFH
			EPN1
			APOBEC3G
			RPL27A
			PPIAL4D
			PCDHA9
			TMTC1
			SMARCD2
			ABCC3
			ATP2C2
			HS6ST1
			AP4B1
			RP5-1073O3.5.1
			SAP130
			SLC22A9
			RASEF
			HMOX2
			RAD23B
			SORCS1

		PLXNB2
		UGGT2
		PVRL2
		PRKCD
		DLG1
		OXR1
		NBPF24
		FOXA2
		CCDC63
		JPH4
		SQLE
		AL583842.6.1
		HOXC12
		PTAR1
		POPDC2
		PTPMT1
		FARP1
		PTPRM
		BCAS3
		C9orf102
		SPRYD5
		KAZN
		ASGR2
		TCEB3
		HECTD1
		DAG1

**suspected cancer genes**		
**PG129 dysplasia only**	**PG129 shared**	**PG129 SCC only**
PRIM2	*ITGB4*	*PIKFYVE*
	NOTCH2	*TCL1A*
	TBP	*NOS1*
	RELN	*PER1*
		FLT1
		SYNE1

**other genes/genomic features**		
**PG129 dysplasia only**	**PG129 shared**	**PG129 SCC only**
PCGF6	*PPM1L*	*XIRP1*
FOXE1	*DDX19B*	*PCSK1*
TRIM5	*RP11-529K1.2.1*	*CTD-2337A12.1.1*
RP11-588H23.3.1	*RP11-529K1.3.1*	*SORCS2*
TPRXL	*PLK5*	*TBC1D3G*
SALL1	*MYLK-AS1*	*SRCRB4D*
KCNN3	*FKBP3*	*NOL8*

HRG	*RP11-380D23.1.1*	*RP11-164H13.1.1*
	ENPEP	*LGR4*
	GOLGA8B	*SLC25A16*
	GOLGA8A	*SLC45A2*
	KDM6B	*SMCHD1*
	GALK1	*AC008735.15.1*
	RP11-551L14.1.1	*CNTN3*
	ROBO2	*NBPF11*
	AMPH	*RAB3IP*
	BSCL2	*AP005901.1.1*
	RP11-831H9.16.1	*WDR11*
	IGSF5	*PNPLA8*
	B4GALT3	*DBT*
	PPOX	*TSNAXIP1*
	DNAH12	*DPP4*
	KCNA7	*AC058791.2.1*
	LILRB3	*RPAP3*
	LILRA6	*LGALS9B*
	RPS9	*RP11-413E6.5.1*
	KLHL6	*STK38L*
	AEBP1	*STK38L*
	LRRC7	*NBPF24*
	AC008103.5.1	*BOD1L*
	BCRP7	*TIPARP*
		CDK14
		RP5-862P8.2.1
		RP11-707M1.1.1
		TEPP
		TRIM24
		NMNAT2

**suspected cancer genes**		
**PG136 dysplasia only**	**PG136 shared**	**PG136 SCC only**
	ITGA2	*BRIP1*
		CYLD

**other genes/genomic features**		
**PG136 dysplasia only**	**PG136 shared**	**PG136 SCC only**
KRTAP10-2	*SCAF8*	*CCT8L1P*
PRSS37	*LRP1*	*EIF4G3*
GLYATL1	*RP11-405A12.2.1*	*BNIP2*
RPL7P41	*GSTO1*	*SMURF1*
TMPRSS13	*TNFRSF11A*	*PCNX*
UBXN11	*CHAF1B*	*ADAP1*
POTEB	*ST6GALNAC3*	*COX19*

PRAMEF6	*ASGR1*	*CCDC91*
NHLRC3	*ANXA7*	*MKLN1*
NBPF20	*CD8B*	*C1orf87*
GNRH2	*SACM1L*	*HDGFL1*
IRF5	*C19orf51*	*VAX1*
	COL18A1	*C10orf126*
	CYP2D7P1	*FBN1*
	ICOS	*DPP4*
	AC138517.4.1	*DDX41*
	PPP4R1L	*CHERP*
	CTD-2009A10.1.1	*CALR3*
		MIPEP
		ATG2B
		KIAA0913
		MLXIP
		SERHL2
		DLG2
		RBM46
		SPICE1
		HERC6
		ABCA2
		APOA2
		PALLD
		ZNF600
		STX3
		AKAP6
		KLHL38
		CDC42BPB
		WDFY3
		APOBEC3F
		GFPT1
		CDK8

**suspected cancer genes**		
**PG137 dysplasia only**	**PG137 shared**	**PG137 SCC only**
TBP	*TP53*	*MAML2*
	LAMC2	*PIK3C2G*
	RUNX1	*CDC27*
	CLCF1	*NCOA2*
	CACNA1B	*ORC5*
		CACNA1B
		ITPR1

**other genes/genomic features**		
**PG137 dysplasia only**	**PG137 shared**	**PG137 SCC only**

NADSYN1	*ZNF738*	*COPS3*
CTD-2611K5.6.1	*MUC4*	*WDR63*
TFCP2	*EHF*	*FOXD4L2*
AC129778.3.1	*SULT1C4*	*TBC1D3G*
ABHD8	*GUCY2C*	*PHIP*
NXF1	*RP11-174G6.1.1*	*EP400*
NOL8	*AHNAK2*	*C18orf34*
DSPP	*AHNAK2*	*SMARCA2*
ANKRD20A3	*NBPF20*	*NFKBIZ*
	STK35	*MRPL45*
	MZT2A	*EYS*
	MZT2A	*FAM188B*
	ACSM5	*INMT-FAM188B*
	AC006557.1	*PGAP1*
	RP11-385D13.1.1	*RPL12*
	CDRT1	*NFKBIZ*
	GMNC	*ELMOD2*
	ORM2	*ARHGAP18*
	CNPY1	*NBPF10*
	RAI14	*BBS9*
	FKBP9	*TRAK1*
	GGT3P	*NPTN*
	CHD3	*ANKRD36C*
	AHNAK	*PPIAL4D*
	EPHA7	*AFAP1*
	MUC4	*FRG1B*
	SNF8	*VPS8*
	APOBEC1	*SF3B3*
	FTH1	*DPY19L2P1*
	LTV1	*USO1*
	TECPR1	*TACC3*
	TXNDC3	*AC016773.1*
	BX284650.1.1	*PTPN4*
	TNRC6B	*ADAM7*
	OSGIN1	*RP11-561E1.1.1*
	RP11-505K9.4.1	*RP11-624C23.1.1*
	DPP6	*FAM129B*
	FAM55A	*SNX29*
	PRDM12	*CTA-250D10.15.1*
		FOXN3
		TM9SF3

**suspected cancer genes**		
**PG144 dysplasia only**	**PG144 shared**	**PG144 SCC only**
SGK1	*CDC27*	*DGKH*

CASP8	*STAT1*	*FZD3*
NFE2L2	*CSF3R*	*KAT2A*
VWF	*NOTCH1*	*PPP2R2B*
MLL3	*POR*	*FAT1*
		CASP8
		SHH
		NFE2L2
		CACNA1I
		WNT5B
		HIST1H3B
		RBL1
		PDE4D
		TEK
		EML4
		AJUBA
		CDKN2A
		ADORA2A
		TBP
		RIMS2
		MYH9

**other genes/genomic features**		
**PG144 dysplasia only**	**PG144 shared**	**PG144 SCC only**
PFAS	*AC009948.3.1*	*XXyac-YM21GA2.7.1*
ARHGAP28	*TTN*	*AQP7P2*
IRF2BPL	*OR4C5*	*RNF44*
HAVCR2	*PVRL3*	*PET112*
MANBA	*NOP16*	*ACOX1*
C19orf44	*AGAP6*	*AQR*
METTL8	*MYH8*	*NEFH*
PDIA2	*KCNQ5*	*MCOLN3*
UNC13C	*RP11-32B5.1.1*	*FAM65A*
NBEA	*WDR52*	*FAM65A*
DDX19B	*RP11-293B20.2.1*	*COL9A1*
ROBO1	*SLC26A9*	*XYLT1*
RP1-310O13.12.1	*PTPRD*	*AGAP7*
DPP10	*RFX6*	*WBSCR17*
DNAH7	*STON2*	*CTNNA2*
GPR124	*AC126365.1.1*	*GLE1*
DNAH1	*SUV420H1*	*GRK4*
PLXNA2	*SRSF7*	*TM6SF1*
OR1N2	*SLC6A9*	*RP11-382A20.3.1*
GSTM2	*NUCB1*	*CTB-99A3.1.1*
NBPF12	*PRAGMIN.1*	*SLC36A1*
ZNF460	*RIMKLB*	*CTDSPL*

RP11-113A10.1.1	*BSN*	*ACADSB*
ZDHHC17	*RP11-467L19.2.1*	*GSR*
GPR128	*RP11-63P12.2.1*	*CEP63*
SGSM1	*SFI1*	*CTD-2228K2.2.1*
CORO1A	*CLVS2*	*AHRR*
GPR98	*TRAPPC4*	*COX18*
SMG1	*NEFH*	*WDFY3*
ZCCHC8	*PRDM2*	*WDFY3-AS1*
CCDC81	*PHB2*	*BET1L*
	EMG1	*METTL14*
	FEZF2	*MEGF8*
	PHF12	*CEP72*
	ATP2C1	*TPPP*
	KLHL24	*MARK4*
	DCDC5	*ZNF462*
	CCR5	*TYR*
	TRAF3IP2	*ARSK*
	TRAF3IP2-AS1	*DUSP19*
	TRAF3IP2	*AC064871.3.1*
	TRAF3IP2-AS1	*TRIM24*
	MIA3	*ESR2*
	NOMO3	*RP11-152F13.2.1*
	CEBPE	*GRSF1*
	ART3	*FPGS*
	ADAM29	*BAHCC1*
		LRGUK
		ANKRD35
		HEATR7B2
		RPS6KC1
		RP11-298I3.5.1
		NADKD1
		PTPRF
		WASF3
		EFR3B
		GFRA1
		GAD1
		CTA-134P22.2.1
		DARC
		NBPF20
		RRM1
		PANK2
		PHLDB2
		SCARF1
		GRB14
		AUTS2
		RPUSD2

		SLC35D1
		PPP4R1L

**suspected cancer genes**		
**PG174 dysplasia only**	**PG174 shared**	**PG174 SCC only**
IST1	*AJUBA*	*LAMA5*
C7	*COL5A1*	*IL1R1*
	CSMD3	*NPY5R*
	FLG	*CDKN2A*
	GNA14	*CACNA1A*
	PLCE1	*CLTC*
	TP53	*ROCK1*
	RNF43	

**other genes/genomic features**		
**PG174 dysplasia only**	**PG174 shared**	**PG174 SCC only**
DDX11	*USH1C*	*ARHGEF40*
NEFH	*CCNL2*	*OSBPL6*
POLR3GL	*MYL1*	*RGL4*
ANKDD1A	*VN1R2*	*GUSBP11*
C14orf23	*SLC6A3*	*ARHGAP40*
MST1	*STK11IP*	*DPP6*
KRTAP4-5	*CCR10*	*CCDC57*
PI4KAP2	*OR4C3*	*NEFH*
	C16orf62	*RP3-377D14.1.1*
	UNC13A	*PRAMEF5*
	ZNF618	*ARMC4*
	DAO	*ZNF704*
	LARGE	*ASGR1*
	IGSF3	*SCAF8*
	RP11-764K9.4.1	*AQR*
	PPM1H	*BMP2K*
	TRBV6-7	*ADAM9*
	GALM	*FAM40B*
	LGR6	*MUC4*
	OR9G1	*EPG5*
	KANK1	*HABP4*
	MEI1	*UBE3C*
	PCDH17	*PMS2CL*
	ZNF446	*MSH3*
	PRDM10	*PCDHB15*
	DUOXA2	*SH2B2*
	DUOX2	*DDX20*
	APOB	*CPNE6*
	CCDC154	*ABAT*

	PI4KAP1	*SV2B*
		FOXK2
		MYH3
		TNRC18C
		TACC3
		AC016773.1
		ANKRD18A
		PRTG
		RP11-420M1.2.1
		RP1-130H16.18.1
		GATSL3
		SLC35D1
		CRYL1
		AL450992.2.1
		FOXK2
		FGD6
		PTPRT

**suspected cancer genes**		
**PG187 dysplasia only**	**PG187 shared**	**PG187 SCC only**
TBP	*CANT1*	*TBP*
	ZFHX4	*CSMD3*
	TP53	
	MYH11	
	LRP5	
	PBRM1	

**other genes/genomic features**		
**PG187 dysplasia only**	**PG187 shared**	**PG187 SCC only**
C1orf9	*MUC12*	*ZBTB22*
GSG1L	*USP31*	*TP53INP1*
GABRB3	*PPM1E*	*ITSN2*
NEFH	*TRIM48*	*KLHL2*
TUBA1B	*STAB2*	*NEBL*
FRMD4A	*TBKBP1*	*C9orf123*
NUP153	*FAM194B*	*MYO1B*
	MYH6	*TERF1P5*
	HIST1H2BD	*RP11-284H19.1.1*
	BRD1	*EIF5A*
	RGS7BP	*FAN1*
	TINAG	*GRM6*
	IFT122	*RP11-281O15.4.1*
	LPHN2	*ANO1*
	COQ6	*THEG*
	DST	*SERHL*

	ZNF738	*FAM151A*
	AC027763.2.1	*ACOT11*
	ZNF704	*KIAA0368*
	IGHV1-8	*CDR2*
		RRN3P3
		GOLGA8A
		RERGL
		RP13-221M14.1.1
		KIR2DL3
		ASB16
		MRVI1
		SLC43A2
		ZP3
		SDHAP1
		CKAP2
		DMC1
		TWF1
		HAGH
		ANKRD20A3
		VPS54
		STK31
		AC131180.1
		CNTROB
		CCT5
		AHNAK2
		PTPRN2
		KCTD1
		DTWD2

**suspected cancer genes**		
**PG192 dysplasia only**	**PG192 shared**	**PG192 SCC only**
	RANBP17	*CHN1*
		KCTD8

**other genes/genomic features**		
**PG192 dysplasia only**	**PG192 shared**	**PG192 SCC only**
OTUD4	*PARP16*	*MPND*
ANKRD36C	*AC104809.3.1*	*GAS2L2*
OR2L3	*ANXA2*	*PNPLA7*
ANKRD18B	*RP11-22B23.1.1*	*SLFN13*
BMS1	*RP11-830F9.6.1*	*DDX11*
UNC13C	*GOLGA8J*	*NBPF12*
UBE2S	*SNHG5*	*SLFN5*
ADAMTS2	*POLR3A*	*ZNF717*
PARP8	*CREBZF*	*RGL4*

FAHD2A	PRH2	GUSBP11
POLR2M	OR3A3	WDR62
GCOM1	TJP1	B4GALT7
KRT10	NBEA	WASF2
RP11-356C4.4.1	GOLGA8J	MUC4
DDX25	LTBR	RP11-830F9.6.1
PRR4	CCDC144NL	U66061.38.1
FCGR2A	MED15	TUBGCP6
MYCBP2	AC006557.1	CORO7
	OR52E4	AMY2A
	NBPF1	DDX31
	PDXDC2P	CASC3
	RP11-106J23.2.1	SLFN5
	UTP3	AC012414.1
	IGHV3-74	UBXN11
	TRGV4	DHX16
	BRD9	CNTNAP3
	ZDHHC11	NBPF11
	TNRC18C	TMEM132B
	CCDC74B	CYTL1
	RP11-823P9.1.1	MPHOSPH8
	ANKRD30B	POU6F2
	PRDM13	SLFN12
	TRAP1	DDX20
	RHPN1	PSG7
	UGT2B28	NBPF3
	GPR172A	ZNF676
	APBA1	MUC20
	IGHV3-74	PSG10P
	GOLGA8J	MUC4
		ANKRD30B
		SLC39A11
		RP11-247C2.1.1
		GAS2L2
		FOXK2
		LDB2
		KRT8
		AC114737.4.1
		IGHGP
		CORO1B
		HOMEZ
		HAUS8

Table S4 – GO terms significantly enriched from lists of genes. For each patient, the filtered genes mutated only in the dysplasia sample, only in the SCC sample, and shared between the samples were tested.

PG004 dysplasia only	PG004 shared	PG004 SCC only
GO:0005524~ATP binding	GO:0016887~ATPase activity	GO:0006366~transcription from RNA polymerase II promoter
GO:0032559~adenyl ribonucleotide binding	GO:0040029~regulation of gene expression, epigenetic	GO:0008188~neuropeptide receptor activity
GO:0030554~adenyl nucleotide binding	GO:0030286~dynein complex	GO:0042923~neuropeptide binding
GO:0001883~purine nucleoside binding	GO:0042995~cell projection	
GO:0001882~nucleoside binding	GO:0001882~nucleoside binding	
GO:0000166~nucleotide binding	GO:0030424~axon	
GO:0032555~purine ribonucleotide binding	GO:0005524~ATP binding	
GO:0032553~ribonucleotide binding	GO:0032559~adenyl ribonucleotide binding	
GO:0017076~purine nucleotide binding	GO:0043005~neuron projection	
	GO:0030554~adenyl nucleotide binding	
	GO:0003810~protein-glutamine gamma-glutamyltransferase activity	
	GO:0001883~purine nucleoside binding	
	GO:0003777~microtubule motor activity	
	GO:0007398~ectoderm development	
	GO:0006349~genetic imprinting	
	GO:0007155~cell adhesion	
	GO:0022610~biological adhesion	
	GO:0045596~negative regulation of cell differentiation	
	GO:0030030~cell projection organization	
	GO:0005887~integral to plasma membrane	
	GO:0005875~microtubule associated complex	
	GO:0043232~intracellular non-membrane-bounded organelle	
	GO:0043228~non-membrane-bounded organelle	
	GO:0060429~epithelium development	
**PG038 dysplasia only**	**PG038 shared**	**PG038 SCC only**
	GO:0000267~cell fraction	GO:0009611~response to wounding
	GO:0005626~insoluble fraction	GO:0035085~cilium axoneme
	GO:0006917~induction of apoptosis	GO:0004252~serine-type endopeptidase activity
	GO:0012502~induction of programmed cell death	
	GO:0006915~apoptosis	
	GO:0012501~programmed cell death	
	GO:0001701~in utero embryonic development	
	GO:0007155~cell adhesion	
	GO:0022610~biological adhesion	
	GO:0006919~activation of caspase activity	
	GO:0008219~cell death	
	GO:0043065~positive regulation of apoptosis	
	GO:0016265~death	
	GO:0043068~positive regulation of programmed cell death	
	GO:0010942~positive regulation of cell death	

	GO:0010952~positive regulation of peptidase activity<br>GO:0043280~positive regulation of caspase activity<br>GO:0042981~regulation of apoptosis<br>GO:0005624~membrane fraction<br>GO:0043067~regulation of programmed cell death<br>GO:0010941~regulation of cell death<br>GO:0043281~regulation of caspase activity<br>GO:0052548~regulation of endopeptidase activity	
**PG049 dysplasia only**	**PG049 shared**	**PG049 SCC only**
		GO:0003044~regulation of systemic arterial blood pressure mediated by a chemical signal<br>GO:0008637~apoptotic mitochondrial changes<br>GO:0007160~cell-matrix adhesion<br>GO:0042826~histone deacetylase binding<br>GO:0005509~calcium ion binding<br>GO:0003073~regulation of systemic arterial blood pressure<br>GO:0031589~cell-substrate adhesion<br>GO:0016529~sarcoplasmic reticulum<br>GO:0016528~sarcoplasm<br>GO:0007568~aging<br>GO:0042149~cellular response to glucose starvation<br>GO:0044420~extracellular matrix part<br>GO:0031669~cellular response to nutrient levels<br>GO:0046907~intracellular transport<br>GO:0030705~cytoskeleton-dependent intracellular transport<br>GO:0006518~peptide metabolic process<br>GO:0051336~regulation of hydrolase activity<br>GO:0001666~response to hypoxia<br>GO:0010149~senescence<br>GO:0007005~mitochondrion organization<br>GO:0070482~response to oxygen levels<br>GO:0006816~calcium ion transport<br>GO:0055093~response to hyperoxia<br>GO:0048147~negative regulation of fibroblast proliferation<br>GO:0031668~cellular response to extracellular stimulus<br>GO:0007346~regulation of mitotic cell cycle<br>GO:0006886~intracellular protein transport<br>GO:0043434~response to peptide hormone stimulus
**PG063 dysplasia only**	**PG063 shared**	**PG063 SCC only**
		GO:0008219~cell death<br>GO:0016265~death<br>GO:0006915~apoptosis<br>GO:0012501~programmed cell death<br>GO:0030182~neuron differentiation<br>GO:0005086~ARF guanyl-nucleotide exchange factor activity

**PG079 dysplasia only**	**PG079 shared**	**PG079 SCC only**
	GO:0007242~intracellular signaling cascade	GO:0051726~regulation of cell cycle<br>GO:0009314~response to radiation<br>GO:0007155~cell adhesion<br>GO:0022610~biological adhesion
**PG105 dysplasia only**	**PG105 shared**	**PG105 SCC only**
	GO:0044430~cytoskeletal part<br>GO:0005856~cytoskeleton<br>GO:0005815~microtubule organizing center<br>GO:0043228~non-membrane-bounded organelle<br>GO:0043232~intracellular non-membrane-bounded organelle<br>GO:0005874~microtubule	
**PG108 dysplasia only**	**PG108 shared**	**PG108 SCC only**
	GO:0045893~positive regulation of transcription, DNA-dependent<br>GO:0051254~positive regulation of RNA metabolic process<br>GO:0003682~chromatin binding<br>GO:0045941~positive regulation of transcription<br>GO:0030518~steroid hormone receptor signaling pathway<br>GO:0010628~positive regulation of gene expression<br>GO:0043414~biopolymer methylation<br>GO:0045935~positive regulation of nucleobase, nucleoside, nucleotide and nucleic acid metabolic process<br>GO:0051173~positive regulation of nitrogen compound metabolic process<br>GO:0035257~nuclear hormone receptor binding<br>GO:0030522~intracellular receptor-mediated signaling pathway<br>GO:0032259~methylation<br>GO:0010557~positive regulation of macromolecule biosynthetic process<br>GO:0031328~positive regulation of cellular biosynthetic process<br>GO:0051427~hormone receptor binding<br>GO:0009891~positive regulation of biosynthetic process<br>GO:0006357~regulation of transcription from RNA polymerase II promoter<br>GO:0000122~negative regulation of transcription from RNA polymerase II promoter<br>GO:0008134~transcription factor binding<br>GO:0010629~negative regulation of gene expression<br>GO:0006730~one-carbon metabolic process<br>GO:0010604~positive regulation of macromolecule metabolic process<br>GO:0003712~transcription cofactor activity<br>GO:0045892~negative regulation of transcription, DNA-dependent<br>GO:0051253~negative regulation of RNA metabolic process<br>GO:0045944~positive regulation of transcription from RNA polymerase II promoter<br>GO:0016571~histone methylation<br>GO:0007242~intracellular signaling cascade	GO:0006351~transcription, DNA-dependent<br>GO:0032774~RNA biosynthetic process<br>GO:0022604~regulation of cell morphogenesis<br>GO:0043232~intracellular non-membrane-bounded organelle<br>GO:0043228~non-membrane-bounded organelle<br>GO:0051130~positive regulation of cellular component organization<br>GO:0046982~protein heterodimerization activity

PG122 dysplasia only	PG122 shared	PG122 SCC only
	GO:0048878~chemical homeostasis	GO:0016447~somatic recombination of immunoglobulin gene segments<br>GO:0016445~somatic diversification of immunoglobulins<br>GO:0008134~transcription factor binding<br>GO:0007507~heart development<br>GO:0002562~somatic diversification of immune receptors via germline recombination within a single locus<br>GO:0016444~somatic cell DNA recombination<br>GO:0002200~somatic diversification of immune receptors<br>GO:0002377~immunoglobulin production<br>GO:0060341~regulation of cellular localization<br>GO:0002440~production of molecular mediator of immune response<br>GO:0002520~immune system development<br>GO:0001775~cell activation
**PG123 dysplasia only**	**PG123 shared**	**PG123 SCC only**
GO:0010558~negative regulation of macromolecule biosynthetic process<br>GO:0031327~negative regulation of cellular biosynthetic process<br>GO:0009890~negative regulation of biosynthetic process<br>GO:0010605~negative regulation of macromolecule metabolic process<br>GO:0005829~cytosol<br>GO:0007568~aging<br>GO:0001701~in utero embryonic development<br>GO:0045596~negative regulation of cell differentiation		GO:0006816~calcium ion transport<br>GO:0050839~cell adhesion molecule binding<br>GO:0050767~regulation of neurogenesis<br>GO:0005626~insoluble fraction<br>GO:0015674~di-, tri-valent inorganic cation transport<br>GO:0016337~cell-cell adhesion<br>GO:0030324~lung development<br>GO:0030323~respiratory tube development<br>GO:0051960~regulation of nervous system development<br>GO:0060541~respiratory system development<br>GO:0060284~regulation of cell development<br>GO:0000267~cell fraction<br>GO:0005624~membrane fraction<br>GO:0014701~junctional sarcoplasmic reticulum membrane<br>GO:0035295~tube development<br>GO:0007406~negative regulation of neuroblast proliferation<br>GO:0001889~liver development<br>GO:0001666~response to hypoxia<br>GO:0043566~structure-specific DNA binding<br>GO:0009416~response to light stimulus<br>GO:0070482~response to oxygen levels<br>GO:0042592~homeostatic process<br>GO:0045765~regulation of angiogenesis<br>GO:0030003~cellular cation homeostasis<br>GO:0019898~extrinsic to membrane<br>GO:0006873~cellular ion homeostasis<br>GO:0042383~sarcolemma<br>GO:0055082~cellular chemical homeostasis
**PG129 dysplasia only**	**PG129 shared**	**PG129 SCC only**

	GO:0044459~plasma membrane part<br>GO:0051057~positive regulation of small GTPase mediated signal transduction<br>GO:0046982~protein heterodimerization activity<br>GO:0008235~metalloexopeptidase activity	GO:0030554~adenyl nucleotide binding<br>GO:0001883~purine nucleoside binding<br>GO:0048471~perinuclear region of cytoplasm<br>GO:0001882~nucleoside binding<br>GO:0042692~muscle cell differentiation<br>GO:0001666~response to hypoxia<br>GO:0070482~response to oxygen levels<br>GO:0000166~nucleotide binding<br>GO:0005524~ATP binding<br>GO:0032559~adenyl ribonucleotide binding<br>GO:0017076~purine nucleotide binding<br>GO:0042277~peptide binding
**PG136 dysplasia only**	**PG136 shared**	**PG136 SCC only**
GO:0004252~serine-type endopeptidase activity	GO:0009897~external side of plasma membrane<br>GO:0005887~integral to plasma membrane<br>GO:0031226~intrinsic to plasma membrane<br>GO:0012505~endomembrane system<br>GO:0006970~response to osmotic stress<br>GO:0009986~cell surface<br>GO:0005509~calcium ion binding<br>GO:0009628~response to abiotic stimulus<br>GO:0006898~receptor-mediated endocytosis	GO:0002697~regulation of immune effector process<br>GO:0048471~perinuclear region of cytoplasm<br>GO:0006886~intracellular protein transport<br>GO:0046907~intracellular transport<br>GO:0032371~regulation of sterol transport<br>GO:0032374~regulation of cholesterol transport<br>GO:0034613~cellular protein localization<br>GO:0070727~cellular macromolecule localization<br>GO:0015031~protein transport
**PG137 dysplasia only**	**PG137 shared**	**PG137 SCC only**
	GO:0005524~ATP binding<br>GO:0032559~adenyl ribonucleotide binding<br>GO:0030554~adenyl nucleotide binding<br>GO:0001883~purine nucleoside binding<br>GO:0001882~nucleoside binding<br>GO:0032555~purine ribonucleotide binding<br>GO:0032553~ribonucleotide binding<br>GO:0000166~nucleotide binding<br>GO:0017076~purine nucleotide binding<br>GO:0043525~positive regulation of neuron apoptosis<br>GO:0010332~response to gamma radiation	GO:0035091~phosphoinositide binding<br>GO:0043232~intracellular non-membrane-bounded organelle<br>GO:0043228~non-membrane-bounded organelle<br>GO:0006886~intracellular protein transport<br>GO:0034613~cellular protein localization<br>GO:0070727~cellular macromolecule localization<br>GO:0005543~phospholipid binding
**PG144 dysplasia only**	**PG144 shared**	**PG144 SCC only**
GO:0048870~cell motility<br>GO:0051674~localization of cell<br>GO:0006928~cell motion<br>GO:0005886~plasma membrane<br>GO:0007218~neuropeptide signaling pathway<br>GO:0005858~axonemal dynein complex<br>GO:0001539~ciliary or flagellar motility<br>GO:0044447~axoneme part	GO:0043232~intracellular non-membrane-bounded organelle<br>GO:0043228~non-membrane-bounded organelle<br>GO:0005730~nucleolus	GO:0000226~microtubule cytoskeleton organization<br>GO:0007017~microtubule-based process<br>GO:0048585~negative regulation of response to stimulus<br>GO:0043005~neuron projection<br>GO:0016331~morphogenesis of embryonic epithelium<br>GO:0070271~protein complex biogenesis<br>GO:0006461~protein complex assembly<br>GO:0065003~macromolecular complex assembly

GO:0035085~cilium axoneme		GO:0002009~morphogenesis of an epithelium<br><br>GO:0007010~cytoskeleton organization<br><br>GO:0043933~macromolecular complex subunit organization<br>GO:0001841~neural tube formation<br><br>GO:0050868~negative regulation of T cell activation<br><br>GO:0001838~embryonic epithelial tube formation<br><br>GO:0042995~cell projection<br><br>GO:0035148~tube lumen formation<br><br>GO:0007267~cell-cell signaling<br><br>GO:0008285~negative regulation of cell proliferation<br><br>GO:0051250~negative regulation of lymphocyte activation<br>GO:0005856~cytoskeleton<br><br>GO:0002695~negative regulation of leukocyte activation<br>GO:0005739~mitochondrion<br><br>GO:0050866~negative regulation of cell activation<br><br>GO:0016337~cell-cell adhesion<br><br>GO:0060562~epithelial tube morphogenesis<br><br>GO:0021915~neural tube development<br><br>GO:0030424~axon<br><br>GO:0006351~transcription, DNA-dependent<br><br>GO:0032774~RNA biosynthetic process<br><br>GO:0043068~positive regulation of programmed cell death<br>GO:0015630~microtubule cytoskeleton<br><br>GO:0010942~positive regulation of cell death<br><br>GO:0048708~astrocyte differentiation<br><br>GO:0060134~prepulse inhibition<br><br>GO:0043025~cell soma<br><br>GO:0048729~tissue morphogenesis<br><br>GO:0050660~FAD binding<br><br>GO:0046636~negative regulation of alpha-beta T cell activation
**PG174 dysplasia only**	**PG174 shared**	**PG174 SCC only**
	GO:0007186~G-protein coupled receptor protein signaling pathway<br>GO:0005886~plasma membrane<br><br>GO:0019725~cellular homeostasis<br><br>GO:0030003~cellular cation homeostasis<br><br>GO:0042592~homeostatic process<br><br>GO:0048878~chemical homeostasis<br><br>GO:0055080~cation homeostasis<br><br>GO:0007204~elevation of cytosolic calcium ion concentration<br>GO:0051480~cytosolic calcium ion homeostasis<br><br>GO:0019957~C-C chemokine binding<br><br>GO:0016493~C-C chemokine receptor activity<br><br>GO:0006873~cellular ion homeostasis<br><br>GO:0055082~cellular chemical homeostasis<br><br>GO:0050801~ion homeostasis	GO:0060341~regulation of cellular localization<br><br>GO:0007010~cytoskeleton organization<br><br>GO:0032300~mismatch repair complex<br><br>GO:0030031~cell projection assembly<br><br>GO:0007160~cell-matrix adhesion<br><br>GO:0030029~actin filament-based process<br><br>GO:0043523~regulation of neuron apoptosis<br><br>GO:0031589~cell-substrate adhesion<br><br>GO:0007155~cell adhesion<br><br>GO:0022610~biological adhesion<br><br>GO:0016337~cell-cell adhesion<br><br>GO:0019899~enzyme binding<br><br>GO:0007268~synaptic transmission<br><br>GO:0042803~protein homodimerization activity

	GO:0004950~chemokine receptor activity<br>GO:0006874~cellular calcium ion homeostasis<br>GO:0019956~chemokine binding	GO:0030155~regulation of cell adhesion<br>GO:0016447~somatic recombination of immunoglobulin gene segments<br>GO:0030983~mismatched DNA binding<br>GO:0007167~enzyme linked receptor protein signaling pathway<br>GO:0019226~transmission of nerve impulse
**PG187 dysplasia only**	**PG187 shared**	**PG187 SCC only**
GO:0000226~microtubule cytoskeleton organization	GO:0048739~cardiac muscle fiber development<br>GO:0032982~myosin filament<br>GO:0005859~muscle myosin complex<br>GO:0016460~myosin II complex<br>GO:0051276~chromosome organization<br>GO:0030239~myofibril assembly<br>GO:0007507~heart development<br>GO:0031032~actomyosin structure organization<br>GO:0030036~actin cytoskeleton organization<br>GO:0015629~actin cytoskeleton<br>GO:0030029~actin filament-based process<br>GO:0048747~muscle fiber development<br>GO:0010927~cellular component assembly involved in morphogenesis	GO:0003779~actin binding<br>GO:0008092~cytoskeletal protein binding
**PG192 dysplasia only**	**PG192 shared**	**PG192 SCC only**
	GO:0005546~phosphatidylinositol-4,5-bisphosphate binding	GO:0070035~purine NTP-dependent helicase activity<br>GO:0008026~ATP-dependent helicase activity<br>GO:0004386~helicase activity<br>GO:0005856~cytoskeleton<br>GO:0043232~intracellular non-membrane-bounded organelle<br>GO:0043228~non-membrane-bounded organelle<br>GO:0042623~ATPase activity, coupled<br>GO:0016887~ATPase activity<br>GO:0007010~cytoskeleton organization
